# Proteomic analysis and cross species comparison of casein fractions from the milk of dairy animals

**DOI:** 10.1038/srep43020

**Published:** 2017-02-27

**Authors:** Xiaxia Wang, Xiaowei Zhao, Dongwei Huang, Xiaocheng Pan, Yunxia Qi, Yongxin Yang, Huiling Zhao, Guanglong Cheng

**Affiliations:** 1 Institute of Animal Science and Veterinary Medicine, Anhui Academy of Agricultural Sciences, Hefei 230031, China

## Abstract

Casein micelles contribute to the physicochemical properties of milk and may also influence its functionality. At present, however, there is an incomplete understanding of the casein micelle associated proteins and its diversity among the milk obtained from different species. Therefore, milk samples were collected from seven dairy animals groups, casein fractions were prepared by ultracentrifugation and their constituent proteins were identified by liquid chromatography tandem mass spectrometry. A total of 193 distinct proteins were identified among all the casein micelle preparations. Protein interaction analysis indicated that caseins could interact with major whey proteins, including β-lactoglobulin, α-lactalbumin, lactoferrin, and serum albumin, and then whey proteins interacted with other proteins. Pathway analysis found that the peroxisome proliferator-activated receptor signaling pathway is shared among the studied animals. Additionally, galactose metabolism pathway is also found to be commonly involved for proteins derived from camel and horse milk. According to the similarity of casein micelle proteomes, two major sample clusters were classified into ruminant animals (Holstein and Jersey cows, buffaloes, yaks, and goats) and non-ruminants (camels and horses). Our results provide new insights into the protein profile associated with casein micelles and the functionality of the casein micelle from the studied animals.

The colloidal casein–calcium–transport complexes known as casein micelles are central to the milk system that play a crucial role in milk synthesis and secretory process^1^. Firstly, casein association and micelle formation may prevent calcium phosphate precipitation and amyloid fibril formation in the mammary gland^2^,^3^. Secondly, casein micelles may also contribute to the physicochemical properties of milk as well as to the stability of the milk and dairy products. Moreover, milk delivers a very high concentration of protein to the neonate, and in large part, this is achieved by packaging caseins into micelles^4^. For these reasons, composition, structure and functions of casein micelles were widely investigated for many years^1^,^4^,^5^.

Previous studies indicated that the ultrastructure of casein micelles is similar, but the micelles differ in composition, size, and hydration in most species^1^,^6^. It is well known that caseins are consist of at least 3 and normally 4 gene products and further divided into α_s1_-casein, α_s2_-casein, β-casein, and κ-casein in farm animals^3^,^7^. Variations in casein contents of milk are widely present, such as, caseins comprise approximately 80% of the total protein in ruminant milk, but only about 55% of the total protein in horse milk^8^,^9^,^10^; furthermore, the relative proportions of caseins differ widely^11^,^12^,^13^. Bovine milk caseins are composed mainly of equal amounts of β-casein and α_s1_-casein^7^, goat and camel milk contain more β-casein than α_s_-casein and κ-casein^12^, and horse milk caseins comprise mainly equal amounts of β-casein and α_s_-casein^6^. In addition, genetic variants and posttranslational modifications of caseins family were reported by several previous studies^14^,^15^,^16^. These results indicated the casein-type composition was different in most dairy animals that are associated with physicochemical properties of milk.

Casein micelles are also characterized by different sizes in the milk from different dairy animals. The size distribution of casein micelles in camel milk is great; these micelles reach the largest diameter, viz., 380 nm^17^, while the average size of casein micelles in goat is 260 nm^13^, and the size of horse milk micelles exceeds those from bovine milk, with a typical value of 150 nm^6^,^8^. These results demonstrated caseins micelle components and size were different in several dairy animals that may contribute to the functionality of milk and milk process^18^.

Calcium, phosphate, and caseins are the major components of casein micelles from dairy animals that had been widely reported in previous studies^2^,^3^,^19^. Notably, casein micelles have been found to bind to hydroxyapatite^20^, vitamin A^21^, and low molecular weight hydrophobic compounds^22^. In addition, interaction between whey proteins and the casein micelle in acidification and heated milk has been demonstrated by diffusing-wave spectroscopy^23^. Recently, β-lactoglobulin and α-lactalbumin were identified in bovine casein fractions by 2-DE combined with MS approach^24^. In another study, casein micelles from human milk has revealed the presence of 82 distinct proteins^25^. Taken together, it is possible that non-casein proteins are involved in the casein micelles; however, to date, protein components and inter-species complexity of the casein micelles have not yet been well elucidated in dairy animals.

Therefore, the objective of this study was to characterize the micelle-associated proteins in parallel from Holstein and Jersey cows, buffaloes, yaks, goats, camels, and horses based on the sample preparation by ultracentrifugation and protein identification by liquid chromatography tandem mass spectrometry (LC-MS/MS) approach. Constituent proteins of micelles are used to reveal a large and diverse repertoire of micelle-associated proteins, and further may help to provide a better understanding of the physiological significance.

## Material and Methods

### Sample Preparation

In the current study, dairy animals were selected during the postpartum days 30–60. Milk samples were collected from multiparous dairy animals in the following districts: 60 Chinese Holstein cows (*Bos taurus*) in Beijing, 21 Jersey cows (*Bos taurus*) in Hebei, 27 goats (*Capra hircus*) in Shanxi, 21 Bactrian camels (*Camelus bactrianus*) and 18 horses (*Equus caballus*) in Xinjiang, 24 yaks (*Bos grunniens*) in Qinghai, and 21 buffaloes (*Bubalus bubalis*) in Yunnan. For Holstein, Jersey and buffalo, milk somatic cell counts less than 200,000 cells/mL were selected. For the other animal groups, all animals were free disease according to the veterinarian record. Samples from Holstein were placed in a box with ice bag and transferred to the laboratory, and samples from other animals were frozen and placed in the box with dry ice and then transferred to the laboratory.

To investigate the differences in the animals groups, the raw milk samples from individual species were pooled into three fractions. First, each milk sample was centrifuged (3000 × *g*) for 15 min at 4 °C; the fat layer was then removed and the skim milk samples were subsequently centrifuged (100 000 × *g*) for 1 h at 4 °C to separate the whey and casein fractions in a Hitachi CS150GX II ultracentrifuge (Hitachi Koki Co. Ltd., Tokyo, Japan). The casein pellet was then collected, washed and resuspended with cold ultrapure water^24^,^26^. After centrifugation, the pellet was resuspended in lysis buffer containing 50 mM Tris-HCl, pH 7.4, with 4% SDS and periodically vortexed for mixing. Subsequently, the samples were subjected to sonication for 5 min at room temperature, followed by incubation in water for 5 min at 95 °C. Finally, the samples were centrifuged (14 000 × *g*) for 40 min at 25 °C. The supernatant was collected and protein concentration was determined using the BCA protocol.

### Protein Digestion

Two hundred micrograms of each sample was reduced by adding dithiothreitol to a final concentration of 100 mM and incubated at 95 °C for 5 min. After cooled, 200 *μ*L UT buffer (8 M urea and 150 mM Tris-HCl, pH 8.0) was added and mixed, and then transferred into an ultrafiltration tube (10-kDa cutoff, Sartorius, Goettingen, Germany) for centrifuging at 14 000 × *g* for 15 min. The sample was subsequently alkylated by adding 100 *μ*L iodoacetamide solution (50 mM iodoacetamide in UT buffer), followed by incubation for 30 min at room temperature in the dark and centrifugation at 14 000 × *g* for 10 min. Two wash steps with 100 *μ*L UT buffer and 100 *μ*L 25 mM ammonium bicarbonate solution were performed, with centrifugation at 14 000 × *g* for 10 min. Finally, the sample was digested by adding 40 *μ*L trypsin (Promega, Madison, WI, USA) buffer (4 *μ*g trypsin in 25 mM ammonium bicarbonate buffer) and incubated at 37 °C for 16–18 h. The filter unit was transferred to a new tube and centrifuged at 14 000 × *g* for 10 min. The digested peptides were collected as a filtrate, and peptide concentration was quantified using a Nanodrop 2000 spectrophotometer (Wilmington, DE, USA) at OD_280_^27^. Samples were desalted using a C18 solid phase extraction column (66872-U, Sigma, USA), dried in a SpeedVac, and stored at −80 °C.

### Liquid chromatography-tandem mass spectrometry analysis

Peptide mixtures were separated and identified using an EASY-nLC 1000 system coupled with a Q-Exactive (Thermo Fisher Scientific, Waltham, MA, USA). Dried peptides were dissolved in buffer A (0.1% [v/v] formic acid and 2% [v/v] acetonitrile in MilliQ-deionized water). The column was equilibrated for 20 min with 95% (v/v) buffer A. A total of 5 μg of each peptide mixture was loaded onto the trap column (20 mm × 150 *μ*m, 5 *μ*m) using an autosampler and separated on a reverse-phase column (100 mm × 150 *μ*m, 3 *μ*m) with buffer B (84% [v/v] acetonitrile and 0.1% [v/v] formic acid in MilliQ-deionized water) using a segmented gradient at 400 nL/min. Peptides were eluted as follows: 0–45% (v/v) buffer B for 100 min, 45–100% (v/v) buffer B for 8 min, followed by a hold at 100% (v/v) buffer B for 12 min.

The Q-Exactive was set up to perform data acquisition in the positive ion mode for 120 min, with a selected mass range of 300–1800 mass/charge (*m/z*). Resolving power for the Q-Exactive was set as 70 000 for the MS scan and 17 500 for the MS/MS scans at *m*/*z* 200. MS/MS data were acquired using the top 10 most abundant precursor ions with charge ≥2 as determined from the MS scan. These were selected with an isolation window of 2 *m/z* and fragmented via higher energy collisional dissociation with normalized collision energies of 27 eV. The maximum ion injection times of the survey scan and the MS/MS scans were 10 and 60 ms, respectively, and the automatic gain control target values for both scan modes were set at 3E6. Dynamic exclusion of the selected precursor ions was set at 40 s. The underfill ratio was defined as 0.1% on the Q-Exactive mass spectrometer.

### Protein identification

Raw files were analyzed using Maxquant software (version 1.3.0.5)^28^. The peak lists were generated and searched against the in-house UniProt database consisting of *Bovidae, Camelus*, and horse sequences, with 86803, 20368, and 28583 entries, respectively (05–2014). The following search parameters were used: monoisotopic mass; MS/MS tolerance at ±20 ppm; maximum number of two missed cleavage sites allowed for trypsin digests of protein; peptide charges of 2+, 3+, and 4+. The fixed modification was defined as the carbamidomethylation of cysteine; variable modifications were specified as the oxidation of methionine and acetylation of the protein N-terminal and deamination of asparagine and glutamine. The decoy database pattern was set as the reversed version of the target database. All reported data were based on 99% confidence for protein and peptide identification as determined by a false discovery rate of no more than 1%^29^. The “match between runs” option was set at a time window of 2 min. Protein identification required at least two unique peptides.

### Co-immunoprecipitation experiment

For Co-immunoprecipitation experiments, milk whey from Holstein cows was used to perform by the Pierce Co-Immunoprecipitation Kit (Thermo Scientific, 26149) according to the manufacturer’s protocol. Briefly, mouse monoclonal anti β-casein was immobilized and covalently linked to resin for 2 h. Milk whey pre-cleared with the control resin were loaded onto columns containing immobilized antibodies and incubated at 4 °C for overnight. Three biological replicate samples were performed. The pulled-down proteins were analyzed by mass spectrometry. Monoclonal antibodies against β-casein of cows were prepared by the Beijing Protein Institute (Beijing, China).

### Western blot analysis

Skim milk samples were run on the 12% SDS-PAGE by a Electrophoresis apparatus (Bio-Rad, Hercules, CA, USA). The gels were electrotransferred onto the polyvinylidine difluoride (PVDF) using a Mini TransBlot apparatus (Bio-Rad, USA). PVDF membranes were blocked with 3% chickens serum in TBST solution (0.1 M Tris pH 7.4, 0.15 M NaCl, 0.1% Tween 20) and incubated with monoclonal anti-bovine β-casein solution at room temperature for 1 h. Subsequently, the PVDF membrane was washed and immersed in the horseradish peroxidase-coupled goat anti-mouse solution in the dark for 1 h. Finally, the membrane was visualized by diaminobenzidene solution. For ‘far-western blot’, the PVDF membrane was incubated with 10 μg/mL β-casein (C6905, Sigma Chemical Co., St. Louis, MO, USA) solution at room temperature for 1 h before the membrane incubated with monoclonal anti-bovine β-casein solution. The other steps were performed as the procedure of western blot.

### Data analysis

Proteins identified in at least two biological replicate samples from triplicate analyses from each animal group were used for subsequent analysis. Proteins that were uniquely identified in the caseins fractions of Holstein and Jersey cow, yak, buffalo, goat, camel, or horse milk were defined as qualitative differences. The potential function of the identified proteins was analyzed using the Uniprot (www.expasy.org) and the Gene Ontology (GO) database (www.geneontology.org). Identified proteins were imported into the online Search Tool for the Retrieval of Interacting Genes/Proteins (STRING) database (http://string-db.org) for known and predicted protein interactions^30^. In order to minimize the rate of false positives, protein–protein interactions confirmed by experimental study, pathways from curated databases, and reported in abstracts of papers published in PubMed were selected. The interactions comprised both direct (physical) and indirect (functional) associations between proteins.

## Results and Discussion

### Identified proteins in casein micelles from the studied animal groups

A total of 193 proteins were identified across species samples using an LC-MS/MS proteomic approach (Table S1). Of these, subsets of the identified proteins in casein micelles from Holstein cows, buffaloes, Jersey cows, yaks, goats, camels, and horses are listed in Table 1. In our study, the sequence database searched contained information on all the studied animals, and would allow identification of either known proteins (i.e., those present in the database), or homologous proteins sharing identical peptides with related database sequences. In previous studies, large protein databases were used to investigate the protein composition in body fluids^31^,^32^. We found that the number of proteins identified in buffaloes was smaller than that in the other studied animals. This low number of proteins identified in casein micelle of buffalo is likely attributable to the incomplete genomic database available for this species and protein sequences differed from the other studied animals, as only a few studies have been conducted on buffaloes to date^33^,^34^.

Aside from α_s1_-casein, α_s2_-casein, β-casein, and κ-casein, other minor proteins such as sulfhydryl oxidase 1, ubiquitin-40S ribosomal protein S27a, and dolichol-phosphate mannosyltransferase subunit 3 were identified in the casein fractions of the animals sampled. This is result of the fact that casein micelle is an open and dynamic structure, as well as hydrophobicity state, which provide the chance allowing casein micelle to trap and/or interact with whey proteins^35^,^36^. Among them, sulfhydryl oxidase is thought to associate with the casein micelle and was identified in samples from all seven species. Sulfhydryl oxidase is widely found in secretory tissues and is particularly associated with the endoplasmic reticulum (ER) lumen and the Golgi apparatus. Farrell Jr *et al*. reviewed and suggested that introduction of molecular oxygen around the time of milking may activate sulfhydryl oxidase and contribute to finalize the formation of the micelles through surface oriented disulfide bonds of κ-casein^1^. In addition, several minor proteins found in this study, such as azurocidin, spermadhesin-1, and tumor necrosis factor receptor superfamily member 6B (TR6), were not detected in milk whey and milk fat globule membrane compartments in previous studies^32^,^34^,^37^,^38^,^39^. Of them, TR6, a member of the TNF receptor family, is a soluble decoy receptor that involved in the regulation of multiple biological processes, including development, apoptosis and immune response by interaction with TNF ligands including TNF-like ligand 1A, tumor necrosis factor superfamily member 14 and CD95^40^,^41^. Similarly, several non-casein proteins were uniquely identified in human milk casein micelle compared to the identified proteins in whey compartment by LC-MS/MS analysis^25^. These results indicated that specific minor proteins associated with casein micelles may extend protein diversity of casein fractions from the studied animal groups.

### Interaction of proteins in the casein micelles

**Figure 1:**
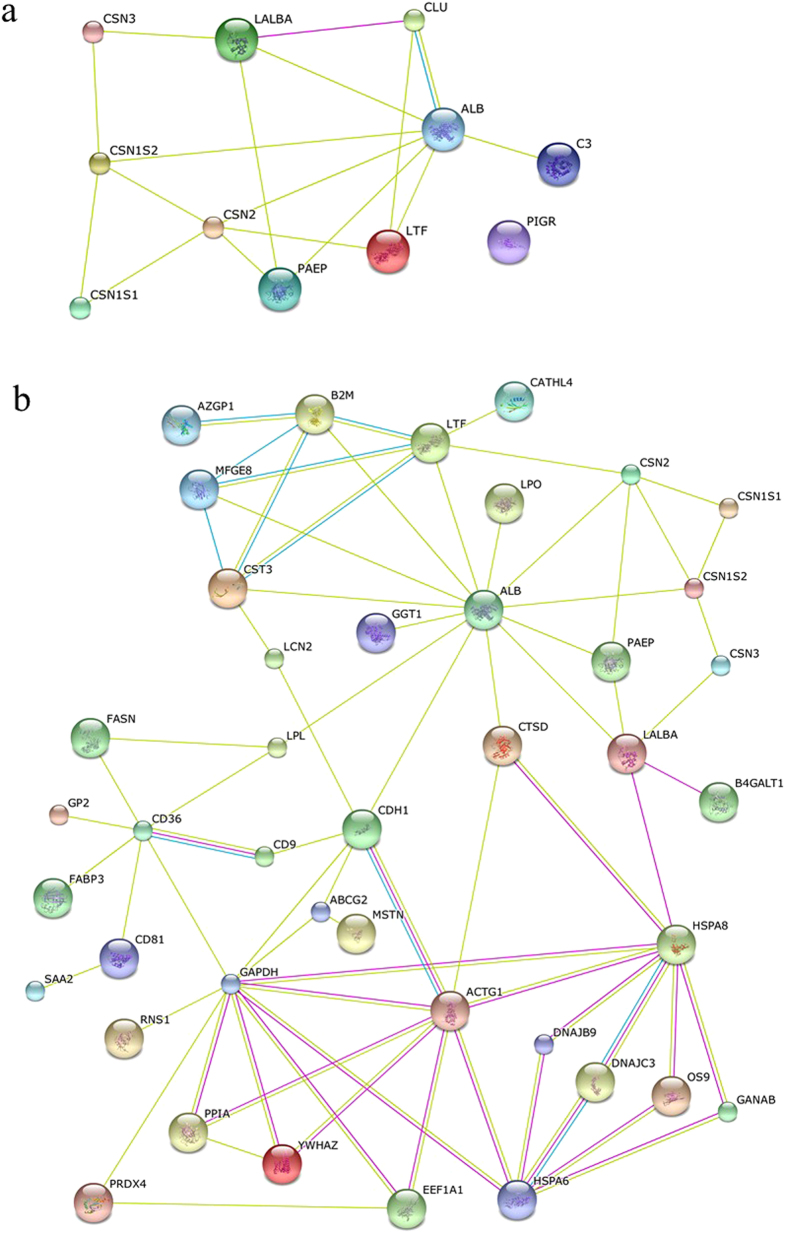
Protein-protein interaction network of the identified proteins from co-immunoprecipitation with antibody β-casein (**a**), casein micelle in Holstein (**b**) generated with STRING software. Each node represents a protein; different line colors represent the types of evidence for the association: pink lines from experimental study, the blue lines from databases, and the yellow lines from abstracts of articles published in PubMed.

**Figure 2:**
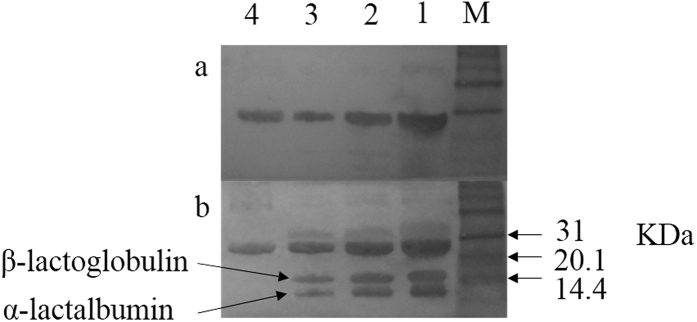
Western blot analysis of β-casein (**a**) and ‘far-western blot’ analysis of β-casein interacted with major whey proteins (**b**). M shows protein marker; Lanes 1, 2, and 3 represent samples with 9, 18 and 36 μg milk proteins; Lanes 4 shows β-casein standard.

To test the interactions between caseins and whey proteins, we performed co-immunoprecipitation experiment using whey proteins and antibody against β-casein. Aside of the β-casein, 13 proteins were identified that may directly and/or indirectly interact with β-casein (Table S2). These proteins were further predicted by STRING software (Fig. 1a). We found that β-casein could interact with α_s1_- and α_s2_-casein, and whey proteins including β-lactoglobulin, lactoferrin, and serum albumin. In addition, interaction of the identified proteins associated with casein micelles from Holstein cows were also predicted by the STRING software and listed in Fig. 1b. Of them, β-casein interacted with whey proteins (β-lactoglobulin and α-lactalbumin) were confirmed by western blot analysis (Fig. 2). Based on these results, we suggested that caseins interact directly with major whey proteins, including β-lactoglobulin, α-lactalbumin, lactoferrin, and serum albumin. These whey proteins then interact with other proteins, including ubiquitin-40S ribosomal protein S27a and β2-microglobulin. Among them, serum albumin may serve as a key node with more relationships than other proteins between caseins and whey proteins that need further investigation.

More recently, a previous study reported that caseins associate with other secreted calcium (phosphate)-binding phosphoproteins, such as osteopontin, in milk^35^. Especially, 82 proteins of the casein micelle in human milk has been identified by LC-MS/MS analysis^25^. In another study, β-lactoglobulin, α-lactalbumin, and serum albumin were identified by 2-DE combined with MS in bovine casein fractions that had been prepared by chymosin-induced separation, isoelectric precipitation, and ultracentrifugation^24^. Taken together, these findings suggested that some non-casein proteins can interact with caseins in milk and form part of the constituents of the casein micelle. These results indicated that caseins can interact with a large number of whey proteins. Thus, the proteins here identified in the casein micelle may contribute to constitute the milk “caseome” from the studied animals. However, further experiments are necessary to confirm biological significance of casein micelles-associated proteins.

### Different proteins in the casein micelles of the studied animal groups

We found that the proteins identified in each animal group differed considerably. Twenty-five proteins were shared in all animal species; 44 proteins were shared in Holstein and Jersey cows, buffaloes, and yaks; and 42 proteins were shared in ruminant animals (Holstein and Jersey cows, buffaloes, yaks, and goats). The results of comparative analysis of casein fractions proteome between species are listed in Table 1. The proteome of Holstein and Jersey cows were most similar, at 83.0% similarity. Overall, the similarity of the “caseome” of ruminant animals (Holstein and Jersey cows, buffaloes, yaks, and goats) was more similar to each other compared with those of non-ruminant animals (camels and horses). Interestingly, our results were in agreement with the previous studies in which several animals were categorized into ruminant and non-ruminant animals group according to their iTRAQ-quantified whey and milk fat globule membrane proteins^34^,^42^.

We also found several proteins that were only identified in Holstein cows, Jersey cows, yaks, buffaloes, goats, camels, and horses, respectively (Table S1). For instance, protein S100-A9 was identified in Holstein cows; ezrin in Jersey cows; odorant-binding protein 2b in yaks; bac7.5 protein and T-complex protein 1 subunit eta in goats; and whey acidic protein, and ferritin heavy chain in camels. Thus, our results distinguished the characteristic traits of the casein micelle from specific species. Several milk whey proteins have previously been reported to allow for identification of milk from different species, such as whey acidic protein and quinone oxidoreductase for camel milk and biglycan for goat milk^34^. Thus, these proteins contribute to the intrinsic characteristic properties of the milk fractions of these species, and may facilitate better understanding of the differences in synthesis and secretion of milk proteins among the studied animals.

### Functional analysis of proteins identified in the casein micelles from the studied animals

**Figure 3:**
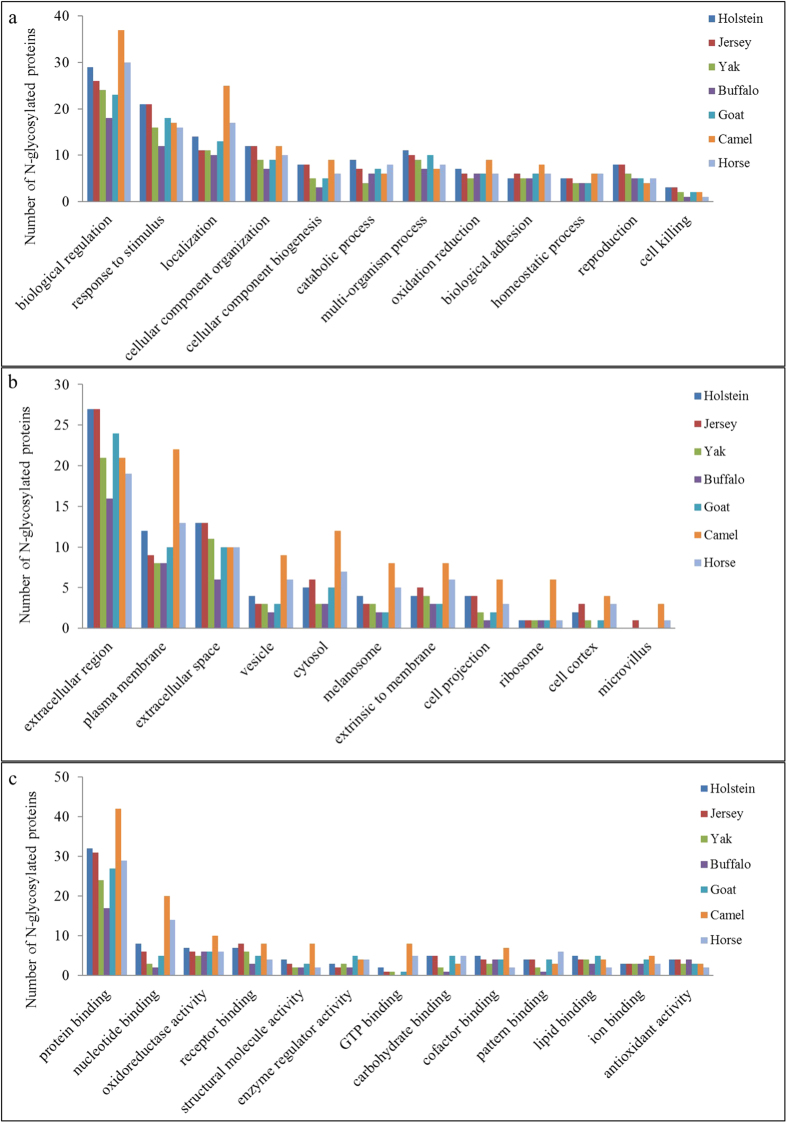
Identified proteins of casein fractions grouped into biological process (**a**), cellular component (**b**) and molecular function (**c**) according to the annotated functions.

To reveal the potential physiological function of the casein micelle, we performed functional analysis of the identified proteins in all the studied animals. All identified proteins were categorized into biological process, cellular component, and molecular function, according to their annotated functions (Fig. 3). The most abundant GO terms in biological processes were biological regulation, response to stimulus, and localization, another major GO terms were cellular component organization, cellular component biogenesis and catabolic process in milk casein fractions from the studied animals. Furthermore, several proteins in milk casein fractions were involved in cell killing. In our previous study, most proteins in the milk whey from Holstein cows, buffaloes, yaks, goats, and camels identified as being involved in biological processes were associated with biological regulation, response to stress, and localization^34^. These results indicated that milk proteins from whey and casein micelles were involved in similar predominant biological processes.

The most abundant GO terms in cellular components were located in the extracellular region,plasma membrane, and extracellular space in milk casein fractions from the studied animals. In addition, several proteins in milk casein fractions were assigned into the vesicle, cytosol, melanosome, and ribosome. We also found that the proteins identified in casein micelle as being involved in cellular component were slightly different among the studied animals. In a previous study, most of the proteins from the human casein micelle were assigned to both extracellular and cytoplasmic locations^25^. These results indicated that specific minor proteins of the casein micelle from different mammals had a slightly different subcellular origin.

The most prevalent molecular functions were binding activity, including protein, nucleotide, receptor, GTP, carbohydrate, cofactor, pattern, lipid and ion binding. Furthermore, several proteins in milk casein fractions were associated with enzyme regulatory, oxidoreductase and structural molecule activity. In a previous study, a larger number of proteins from milk whey were associated with the binding category, and a small number of proteins were involved in various enzyme activities according to their annotations^39^. In another previous study, functional analysis of proteins identified in milk whey from Holstein cows, buffaloes, yaks, goats, and camels demonstrated that the predominant molecular function was binding activity, while another major functional category was enzyme regulatory activity^34^. These results indicated that milk proteins from whey and casein fractions had a similar predominant molecular function.

Pathway analysis of the identified proteins from each animal group are categorized; we found that peroxisome proliferator-activated receptor (PPAR) signaling pathway was the commonly shared pathway in all the aforementioned animals being significantly matched. In addition, ribosome, galactose metabolism, and antigen processing and presentation pathways are also enriched in the camel milk casein fraction, and galactose metabolism is also enriched in the horse milk casein fraction. Enriched pathways with statistical significance are presented in Table 2. In our previous study, we found that several proteins from milk fat globule membrane fractions were assigned to PPAR signaling pathway by iTRAQ proteomic approach^42^. It is well known that PPAR regulate genes involved in lipid metabolism and adipocyte differentiation. Recently, a researcher group found that PPAR pathway was associated with synthesis and secretion milk in the mammary gland throughout lactation by transcriptome analysis^43^,^44^. Thus, our results indicated that some proteins in casein micelles may contribute to milk synthesis and secretion, and protein composition of casein micelles and their relationship with some specific cellular function need further explored.

## Conclusion

In the current study, proteomic analyses of the casein micelles in the milk of Holstein and Jersey cows, yaks, buffaloes, goats, camels, and horses were systematically performed by LC-MS/MS. The results from this study enhance our understanding of the milk “caseome” and its potential physiological functions, as well as help pinpoint interaction networks that may contribute to better understanding of the functionality of the casein micelle.

## Additional Information

**How to cite this article:** Wang, X. *et al*. Proteomic analysis and cross species comparison of casein fractions from the milk of dairy animals. *Sci. Rep.* **7**, 43020; doi: 10.1038/srep43020 (2017).

**Publisher's note:** Springer Nature remains neutral with regard to jurisdictional claims in published maps and institutional affiliations.

## Supplementary Material

Supplementary Information

## Figures and Tables

**Table 1:** Comparative analysis of identified proteins in casein fractions between the studied animals.

	Holstein	Jersey	Yak	Buffalo	Goat	Camel	Horse
Holstein	88	73 (91.3%)	63 (91.3%)	46 (85.2%)	56 (75.7%)	45 (45.0%)	45 (43.7%)
Jersey	73 (83.0%)	80	62 (89.9%)	49 (90.7%)	55 (74.3%)	42 (42.0%)	44 (42.7%)
Yak	63 (71.6%)	62 (77.5%)	69	44 (81.5%)	51 (68.9%)	36 (36.0%)	37 (35.9%)
Buffalo	46 (52.3%)	49 (61.3%)	44 (63.8%)	54	42 (56.8%)	32 (32.0%)	33 (32.0%)
Goat	56 (63.6%)	55 (68.8%)	51 (73.9%)	42 (77.8%)	74	39 (39.0%)	41 (39.8%)
Camel	45 (51.1%)	42 (52.5%)	36 (52.2%)	32 (59.3%)	39 (52.7%)	100	55 (53.4%)
Horse	45 (51.1%)	44 (55.0%)	37 (53.6%)	33 (61.1%)	41 (55.4%)	55 (55.0%)	103

Each value presents the number of identified proteins in casein fraction shared between species (column × line).

**Table 2:** Pathway analysis of identified proteins of casein fractions in Holstein, Jersey, buffalo, yak, goat, camel and horse.

Animal	Pathway Name	Count	Hits	Percent %	*P* Value	Fold Enrichment
Holstein	PPAR signaling pathway	3	67	3. 66	0.0478	8.20
Jersey	PPAR signaling pathway	3	67	4.05	0.0413	8.85
Yak	PPAR signaling pathway	3	67	7.81	0.0002	16.75
Buffalo	PPAR signaling pathway	5	67	5.88	0.0192	13.01
Goat	PPAR signaling pathway	4	67	5.80	0.0040	11.80
Camel	Ribosome	6	84	6.25	0.0011	7.35
	Galactose metabolism	3	24	3.12	0.0214	12.86
	PPAR signaling pathway	4	67	4.17	0.0253	6.14
	Antigen processing and presentation	4	67	4.17	0.0253	6.14
Horse	PPAR signaling pathway	5	67	5.75	0.0008	11.17
	Galactose metabolism	3	24	3.45	0.0103	18.71
