# Supplementary material for: Proteomic analysis and cross species comparison of casein fractions from the milk of dairy animals
Source: Sci Rep. 2017 Feb 27;7:43020. doi: 10.1038/srep43020 (PMC5327394; doi:10.1038/srep43020)
Supplement: Supplementary Information [file srep43020-s1.doc]

**Proteomic analysis and cross species comparison of casein fractions from the milk of dairy animals**

Xiaxia Wang^+^, Xiaowei Zhao^+^, Dongwei Huang, Xiaocheng Pan, Yunxia Qi, Yongxin Yang*, Huiling Zhao, Guanglong Cheng

*Institute of Animal Science and Veterinary Medicine, Anhui Academy of Agricultural Sciences, Hefei 230031, China*

*To whom correspondence should be addressed.

Tel.: +86 551 65146065.

Fax: +86 551 62160275.

E-mail: yyongxin@yahoo.com

Table S1 Identified proteins of casein fractions from seven animal groups.

| Majority protein IDs | Protein name | Gene name | Peptides | Sequence coverage [%] | Mol. Weight (kDa) | | |
| --- | --- | --- | --- | --- | --- | --- | --- |
| **Holstein** |  |  |  |  |  | | |
| A7E3T8;L8IFC5;Q32PJ1 | ATP-binding cassette, sub-family G, member 2 | ABCG2 | 4 | 5.5 | 73.077 | | |
| K9IS26;P63258 | Actin, cytoplasmic 2 | ACTG1 | 13 | 47.1 | 42.273 | | |
| L8ISP4 | Serum albumin | ALB | 26 | 52.2 | 69.337 | | |
| P10152;Q2NKV1;L8I8Q7 | Angiogenin-1 | ANG | 5 | 41.2 | 16.969 | | |
| A6H6X2;P80929 | Angiogenin-2 | ANG2 | 5 | 40.1 | 17.154 | | |
| P46193;Q6BCI9;M3WM96 | Annexin A1 | ANXA1 | 2 | 7.8 | 38.979 | | |
| Q3ZCH5;F1RNP2;L8IS19 | Zinc-alpha-2-glycoprotein | AZGP1 | 3 | 12.4 | 33.851 | | |
| G3N2H4;L8HQG0;G3N0Q8 | Azurocidin | AZU1 | 2 | 14.2 | 28.102 | | |
| P01888;Q862Q3;Q6VUQ8 | Beta-2-microglobulin | B2M | 5 | 55.9 | 13.677 | | |
| P08037;P08037-2;L8I3I4 | Beta-1,4-galactosyltransferase 1 | B4GALT1 | 6 | 27.1 | 44.842 | | |
| P18892;L8HLI3 | Butyrophilin subfamily 1 member A1 | BTN1A1 | 17 | 42.8 | 59.276 | | |
| L8HPZ1;P22226;P54230 | Cathelicidin-1 | CATHL1 | 6 | 43.9 | 17.631 | | |
| P19660;P56425;P54228 | Cathelicidin-2 | CATHL2 | 4 | 25.6 | 20.03 | | |
| P33046 | Cathelicidin-4 | CATHL4 | 3 | 31.2 | 16.479 | | |
| L8IY96;P26201 | Platelet glycoprotein 4 | CD36 | 4 | 11.7 | 52.912 | | |
| Q3ZCD0;L8I3A3 | CD81 antigen | CD81 | 3 | 25 | 25.853 | | |
| P30932;L8HXM8 | CD9 antigen | CD9 | 3 | 20.8 | 25.258 | | |
| A6QLC4;L8IKZ0;Q6R8F2 | Cadherin-1 | CDH1 | 2 | 2.2 | 97.974 | | |
| G3X7D2;I6YIV5;P30922 | Chitinase-3-like protein 1 | CHI3L1 | 4 | 17.4 | 43.737 | | |
| F1MX50;Q148D9 | Cellular repressor of E1A-stimulated genes 1 | CREG1 | 4 | 25.3 | 23.958 | | |
| Q3ZCL0;F6R3I5 | Cysteine-rich secretory protein 2 | CRISP3 | 6 | 36.5 | 27.453 | | |
| A6QLZ7;L8IQ63 | Cysteine-rich secretory protein LCCL domain-containing 2 | CRISPLD2 | 4 | 9.9 | 55.581 | | |
| L8I5S0;B5B3R8 | Alpha-S1-casein | CSN1S1 | 13 | 75.2 | 24.457 | | |
| E9NZN2;B6VPY3 | Alpha-S2-casein | CSN1S2 | 20 | 62.2 | 26.118 | | |
| B7VGH4;Q9TSI0 | Beta casein | CSN2 | 8 | 47.3 | 25.106 | | |
| A8KRP5;P11840;Q712N6 | Kappa-casein | CSN3 | 9 | 45.3 | 21.409 | | |
| L8J0Y3;P01035 | Cystatin-C (Fragment) | CST3 | 4 | 45.7 | 16.651 | | |
| P80209;Q9BGU5 | Cathepsin D | CTSD | 4 | 12.6 | 44.704 | | |
| F7CXT0 | Uncharacterized protein (Fragment) | CYBRD1 | 2 | 11.3 | 24.497 | | |
| G3MZ88;L8I0U1 | DnaJ-like protein subfamily B member 9 | DNAJB9 | 4 | 22 | 25.715 | | |
| F1N036;Q27968 | DnaJ homolog subfamily C member 3 | DNAJC3 | 3 | 7.5 | 57.731 | | |
| G1P1X5;G1MKK3 | Dolichol-phosphate mannosyltransferase subunit 3 | DPM3 | 2 | 19.8 | 12.169 | | |
| G1LMH6;A2Q0Z0 | Elongation factor 1-alpha | EEF1A1 | 9 | 29.2 | 50.856 | | |
| E5G7E7;L8HZH7;P10790 | Fatty acid-binding protein | FABP3 | 8 | 58.6 | 14.779 | | |
| F1N647;Q71SP7 | Fatty acid synthase | FASN | 28 | 16.4 | 274.26 | | |
| Q9MZ06;Q4VS17 | Fibroblast growth factor-binding protein 1 | FGFBP1 | 5 | 18.8 | 26.188 | | |
| L8I843;Q3SZZ9;P12799 | Fibrinogen gamma-B chain | FGG | 5 | 16.5 | 50.231 | | |
| Q0VCN9;Q9XSH1;P02702 | Folate receptor alpha | FOLR1 | 2 | 7.4 | 29.753 | | |
| A6QNJ8;L8I0R2;P79403 | Neutral alpha-glucosidase AB | GANAB | 11 | 14.6 | 109.47 | | |
| A3FKF7;P10096 | Glyceraldehyde-3-phosphate dehydrogenase | GAPDH | 6 | 26.7 | 35.82 | | |
| G3N2D8;L8I7J4;K9J1V5 | Gamma-glutamyltranspeptidase 1 (Fragment) | GGT1 | 2 | 3.7 | 60.904 | | |
| P80195 | Glycosylation-dependent cell adhesion molecule 1 | GLYCAM1 | 2 | 14.4 | 17.151 | | |
| F1N726;L8IK54;Q0IIA4 | Glycoprotein 2 | GP2 | 12 | 26.6 | 59.248 | | |
| F1RPA3;F7B3U1 | G protein-coupled receptor, family C, group 5, member B | GPRC5B | 3 | 8.1 | 45.312 | | |
| E1BHY6;Q2HJI6 | Granulin | GRN | 7 | 15 | 63.081 | | |
| E1BGX8;L8I229 | HHIP-like protein 2 (Fragment) | HHIPL2 | 11 | 16.3 | 81.178 | | |
| L8I940;P62802 | Histone H4 | HI | 4 | 42.7 | 11.305 | | |
| Q04967;E2JF15 | Heat shock 70 kDa protein 6 | HSPA6 | 3 | 6.2 | 71.108 | | |
| P19120;K9J3G1 | Heat shock cognate 71 kDa protein | HSPA8 | 7 | 16.9 | 71.238 | | |
| L8HW39;Q9XSG3 | Isocitrate dehydrogenase [NADP] cytoplasmic | IDH1 | 7 | 21.7 | 47.421 | | |
| A5D7Q2;L8HP94 | Ig alpha-1 chain C region (Fragment) | IGHA1 | 8 | 26.1 | 51.67 | | |
| Q3SYR8 | Immunoglobulin J chain | IGJ | 3 | 21 | 17.857 | | |
| L8IAF4 | Ig lambda-1 chain C regions (Fragment) | IGLC1 | 5 | 74.3 | 11.185 | | |
| L5L6W7;L5MAH8 | Keratin, type II cytoskeletal 75 | KRT75 | 8 | 11.4 | 60.758 | | |
| B1A4S9 | Alpha-lactalbumin | LALBA | 6 | 59.2 | 16.304 | | |
| F1MNN7;Q2TBI0 | Lipopolysaccharide-binding protein | LBP | 5 | 12.9 | 53.694 | | |
| E1B6Z6;L8HVH6 | Neutrophil gelatinase-associated lipocalin | LCN2 | 5 | 36.5 | 22.983 | | |
| P24627;Q19KS1 | Lactotransferrin | LF | 39 | 59.7 | 78.056 | | |
| B5B0D4 | beta-lactoglobulin | LGB | 12 | 73.6 | 19.969 | | |
| Q95JH0;Q95JH2 | Lysophosphatidic acid acyltransferase | LPAAT | 2 | 14.6 | 32.071 | | |
| P11151;Q29524;H9E8W0 | Lipoprotein lipase | LPL | 10 | 28.9 | 53.591 | | |
| L8ICE9;P80025;G3MXZ0 | Lactoperoxidase | LPO | 18 | 34.6 | 80.611 | | |
| Q95114;Q3T0K7;Q95114 | Lactadherin | MFGE8 | 20 | 53.8 | 47.846 | | |
| O18831;Q6SVB3 | Myostatin | MSTN | 4 | 13.1 | 42.778 | | |
| A7E340;E1BGW1;Q8MI01 | Mucin 15, cell surface associated | MUC15 | 3 | 9.4 | 35.685 | | |
| Q05927;L8ICQ2 | 5-nucleotidase | NT5E | 2 | 4.5 | 62.965 | | |
| F1MX65;L8I951;Q3MHX6 | Protein OS-9 | OS9 | 2 | 4.5 | 75.806 | | |
| B5T255;L8IR97 | Peptidoglycan recognition protein 1 | PGLYRP1 | 6 | 39.5 | 21.037 | | |
| P81265;A6QNW3 | Polymeric immunoglobulin receptor | PIGR | 17 | 34.2 | 82.434 | | |
| Q9TUM6;F1MHI1 | Perilipin-2 | PLIN2 | 15 | 45.6 | 49.307 | | |
| P62935;P62936 | Peptidyl-prolyl cis-trans isomerase | PPIA | 2 | 19.5 | 17.77 | | |
| L8I1I1;Q9BGI2 | Peroxiredoxin-4 | PRDX4 | 3 | 20.1 | 30.741 | | |
| A6QQA8;F1MM32 | Sulfhydryl oxidase 1 | QSOX1 | 10 | 23.6 | 62.974 | | |
| L5LW97;G1MC16 | Ras-related protein Rab-18 | RAB18 | 3 | 20.5 | 26.935 | | |
| P61823;P07847 | Ribonuclease pancreatic | RNASE1 | 2 | 28 | 16.477 | | |
| A5PJY7;L8I9S6 | Non-secretory ribonuclease | RNASE2 | 2 | 14.6 | 18.091 | | |
| Q58DP6;L8I6U9;P15467 | Ribonuclease, RNase A family, 4 | RNASE4 | 5 | 50.3 | 16.938 | | |
| P28783;F1MHS5;E1BLI9 | Protein S100-A9 | S100A9 | 2 | 19.2 | 17.114 | | |
| Q32PB7;Q8SQ28 | Serum amyloid A protein (Fragment) | SAA | 5 | 36.1 | 15.897 | | |
| F1N6D4;Q27960 | Sodium-dependent phosphate transport protein 2B | SLC34A2 | 6 | 11.5 | 75.847 | | |
| G8JKX0;L8IL82;P29392 | Spermadhesin-1 | SPADH1 | 2 | 22.4 | 15.022 | | |
| A6QPW7;J9P6Y0 | Tumor necrosis factor receptor superfamily member 6B | TNFRSF6B | 2 | 7.1 | 32.375 | | |
| P0CH28;L8IDB8;P0CG68 | Ubiquitin-40S ribosomal protein S27a | UBC | 4 | 56.1 | 77.569 | | |
| P80457 | Xanthine dehydrogenase/oxidase | XDH | 31 | 32.7 | 146.79 | | |
| K9IR91;P63103 | 14-3-3 protein zeta/delta | YWHAZ | 7 | 34.5 | 29.364 | | |
| G3N0V0 | Uncharacterized protein (Fragment) |  | 5 | 23 | 35.951 | | |
| F1MLW8 | Uncharacterized protein |  | 4 | 21.9 | 24.623 | | |
| G5E604;L8HK92 | Uncharacterized protein |  | 4 | 43.9 | 11.058 | | |
| **Jersey** |  |  |  |  |  | | |
| L5K515;A4IFM8;O62788 | Actin, alpha cardiac muscle 1 | ACTA1 | 8 | 24.1 | 42.975 | | |
| A0S012;F7A5E3;Q7M3B0 | Actin, cytoplasmic 2 | ACTG1 | 13 | 47.1 | 42.273 | | |
| L8ISP4 | Serum albumin | ALB | 26 | 52.2 | 69.337 | | |
| P10152;Q2NKV1;L8I8Q7 | Angiogenin-1 | ANG | 5 | 41.2 | 16.969 | | |
| A6H6X2;P80929 | Angiogenin-2 | ANG2 | 5 | 40.1 | 17.154 | | |
| Q3ZCH5;F1RNP2;L8IS19 | Zinc-alpha-2-glycoprotein | AZGP1 | 3 | 12.4 | 33.851 | | |
| P01888;Q862Q3;L8HKA5 | Beta-2-microglobulin | B2M | 5 | 55.9 | 13.677 | | |
| P08037;P08037-2;L8I3I4 | Beta-1,4-galactosyltransferase 1 | B4GALT1 | 6 | 27.1 | 44.842 | | |
| P18892;L8HLI3 | Butyrophilin subfamily 1 member A1 | BTN1A1 | 17 | 42.8 | 59.276 | | |
| L8HPZ1;P22226;P54230 | Cathelicidin-1 | CATHL1 | 6 | 43.9 | 17.631 | | |
| P33046 | Cathelicidin-4 | CATHL4 | 3 | 31.2 | 16.479 | | |
| M9WLX8 | CD36 | CD36 | 5 | 14.4 | 52.742 | | |
| P30932;L8HXM8;G8JKX6 | CD9 antigen | CD9 | 3 | 20.8 | 25.258 | | |
| A6QLC4;L8IKZ0;Q6R8F2 | Cadherin-1 | CDH1 | 2 | 2.2 | 97.974 | | |
| G3X7D2;I6YIV5;P30922 | Chitinase-3-like protein 1 | CHI3L1 | 4 | 17.4 | 43.737 | | |
| F1MX50;Q148D9 | Cellular repressor of E1A-stimulated genes 1 | CREG1 | 4 | 25.3 | 23.958 | | |
| Q3ZCL0;F6R3I5 | Cysteine-rich secretory protein 2 | CRISP3 | 6 | 36.5 | 27.453 | | |
| A6QLZ7;D1Z306;L8IQ63 | Cysteine-rich secretory protein LCCL domain-containing 2 | CRISPLD2 | 4 | 9.9 | 55.581 | | |
| L8I5S0;B5B3R8 | Alpha-S1-casein | CSN1S1 | 13 | 75.2 | 24.457 | | |
| E9NZN2;B6VPY3 | Alpha-S2-casein | CSN1S2 | 20 | 62.2 | 26.118 | | |
| B7VGH4;Q9TSI0 | Beta casein | CSN2 | 8 | 47.3 | 25.106 | | |
| K9ZS80;K9ZUK9;Q5C9H2 | Kappa casein | CSN3 | 8 | 56.9 | 15.862 | | |
| L8J0Y3;P01035 | Cystatin-C | CST3 | 4 | 45.7 | 16.651 | | |
| P80209;Q9BGU5 | Cathepsin D | CTSD | 4 | 12.6 | 44.704 | | |
| F1MD23;Q2KIE5 | Chemokine (C-X-C motif) ligand 2 | CXCL2 | 2 | 31.7 | 10.998 | | |
| F7CXT0 | Uncharacterized protein (Fragment) | CYBRD1 | 2 | 11.3 | 24.497 | | |
| G3MZ88;L8I0U1 | DnaJ-like protein subfamily B member 9 | DNAJB9 | 4 | 22 | 25.715 | | |
| F1N036;Q27968 | DnaJ homolog subfamily C member 3 | DNAJC3 | 3 | 7.5 | 57.731 | | |
| G1P1X5;Q3ZC71;L5JTX7 | Dolichol-phosphate mannosyltransferase subunit 3 | DPM3 | 2 | 19.8 | 12.169 | | |
| P68103;Q0PY11 | Elongation factor 1-alpha | EEF1A1 | 9 | 29.2 | 50.856 | | |
| P31976;L8IBW5 | Ezrin | EZR | 5 | 12.4 | 68.759 | | |
| P10790;Q4TZH2;Q5XLB1 | Fatty acid-binding protein | FABP3 | 8 | 58.6 | 14.779 | | |
| F1N647;Q71SP7 | Fatty acid synthase | FASN | 28 | 16.4 | 274.26 | | |
| Q9MZ06;Q4VS17 | Fibroblast growth factor-binding protein 1 | FGFBP1 | 5 | 18.8 | 26.188 | | |
| Q3SZZ9;P12799 | Fibrinogen gamma-B chain | FGG | 5 | 16.5 | 50.231 | | |
| A6QNJ8;L8I0R2 | Neutral alpha-glucosidase AB | GANAB | 11 | 14.6 | 109.47 | | |
| G3N2D8;L8I7J4;K9J1V5 | Gamma-glutamyltranspeptidase 1 (Fragment) | GGT1 | 2 | 3.7 | 60.904 | | |
| P80195 | Glycosylation-dependent cell adhesion molecule 1 | GLYCAM1 | 2 | 14.4 | 17.151 | | |
| F1N726;L8IK54;Q0IIA4 | Glycoprotein 2 | GP2 | 12 | 26.6 | 59.248 | | |
| H9GZN9 | Uncharacterized protein (Fragment) | IGHM | 16 | 50.4 | 49.438 | | |
| Q1JPD9;D2HYX4 | G protein-coupled receptor, family C, group 5, member B | GPRC5B | 3 | 8.1 | 45.312 | | |
| E1BHY6;Q2HJI6 | Granulin | GRN | 7 | 15 | 63.081 | | |
| E1BGX8;L8I229 | HHIP-like protein 2 (Fragment) | HHIPL2 | 11 | 16.3 | 81.178 | | |
| Q04967;E2JF15 | Heat shock 70 kDa protein 6 | HSPA6 | 3 | 6.2 | 71.108 | | |
| P19120;K9J3G1 | Heat shock cognate 71 kDa protein | HSPA8 | 7 | 16.9 | 71.238 | | |
| Q9XSG3;Q0QEQ4 | Isocitrate dehydrogenase [NADP] cytoplasmic | IDH1 | 7 | 21.7 | 47.421 | | |
| A5D7Q2;L8HP94;G3MXB5 | Ig alpha-1 chain C region (Fragment) | IGHA1 | 8 | 26.1 | 51.67 | | |
| Q3SYR8 | Immunoglobulin J chain | IGJ | 3 | 21 | 17.857 | | |
| L8IAF4 | Ig lambda-1 chain C regions (Fragment) | IGLC1 | 5 | 74.3 | 11.185 | | |
| F6YRC5;M3W5R5;L5L5G8 | Ras GTPase-activating-like protein IQGAP1 | IQGAP1 | 2 | 2.3 | 187.12 | | |
| L5L6W7;L5MAH8 | Keratin, type II cytoskeletal 75 | KRT75 | 8 | 11.4 | 60.758 | | |
| B1A4S9 | Alpha-lactalbumin | LALBA | 6 | 59.2 | 16.304 | | |
| F1MNN7;Q2TBI0 | Lipopolysaccharide-binding protein | LBP | 5 | 12.9 | 53.694 | | |
| E1B6Z6;L8HVH6 | Neutrophil gelatinase-associated lipocalin | LCN2 | 5 | 36.5 | 22.983 | | |
| P24627;Q19KS1 | Lactotransferrin | LF | 39 | 59.7 | 78.056 | | |
| B5B0D4 | beta-lactoglobulin | LGB | 12 | 73.6 | 19.969 | | |
| Q95JH0;Q95JH2 | Lysophosphatidic acid acyltransferase | LPAAT | 2 | 14.6 | 32.071 | | |
| P11151;Q29524 | Lipoprotein lipase | LPL | 10 | 28.9 | 53.591 | | |
| L8ICE9;P80025;G3MXZ0 | Lactoperoxidase | LPO | 18 | 34.6 | 80.611 | | |
| Q95114;Q3T0K7 | Lactadherin | MFGE8 | 20 | 53.8 | 47.846 | | |
| Q3MHX6;L5M9N2 | Protein OS-9 | OS9 | 2 | 4.5 | 75.806 | | |
| B5T255;L8IR97 | Peptidoglycan recognition protein 1 | PGLYRP1 | 6 | 39.5 | 21.037 | | |
| P81265;A6QNW3 | Polymeric immunoglobulin receptor | PIGR | 17 | 34.2 | 82.434 | | |
| A1L5C2;F1N1N6;Q9TUM6 | Perilipin-2 | PLIN2 | 15 | 45.6 | 49.307 | | |
| P62935;P62936 | Peptidyl-prolyl cis-trans isomerase | PPIA | 2 | 19.5 | 17.77 | | |
| Q9BGI2;K7GLN4 | Peroxiredoxin-4 | PRDX4 | 3 | 20.1 | 30.741 | | |
| A6QQA8;F1MM32 | Sulfhydryl oxidase 1 | QSOX1 | 10 | 23.6 | 62.974 | | |
| L8I703;P61823;P07847 | Ribonuclease pancreatic | RNASE1 | 2 | 28 | 16.477 | | |
| A5PJY7;L8I9S6 | Non-secretory ribonuclease | RNASE2 | 2 | 14.6 | 18.091 | | |
| Q58DP6;L8I6U9;P15467 | Ribonuclease, RNase A family, 4 | RNASE4 | 5 | 50.3 | 16.938 | | |
| Q32PB7;Q8SQ28 | Serum amyloid A protein (Fragment) | SAA | 5 | 36.1 | 15.897 | | |
| Q27960;E9NST3 | Sodium-dependent phosphate transport protein 2B | SLC34A2 | 6 | 11.5 | 75.847 | | |
| G8JKX0;L8IL82;P29392 | Spermadhesin-1 | SPADH1 | 2 | 22.4 | 15.022 | | |
| Q9XSC9;L8HL52 | Transcobalamin-2 | TCN2 | 4 | 10.6 | 47.958 | | |
| A6QPW7;J9P6Y0 | Tumor necrosis factor receptor superfamily member 6B | TNFRSF6B | 2 | 7.1 | 32.375 | | |
| P0CH28;L8IDB8;P0CG68 | Ubiquitin-40S ribosomal protein S27a | UBC | 4 | 56.1 | 77.569 | | |
| P80457 | Xanthine dehydrogenase/oxidase | XDH | 31 | 32.7 | 146.79 | | |
| G3N0V0 | Uncharacterized protein (Fragment) |  | 5 | 23 | 35.951 | | |
| F1MLW8 | Uncharacterized protein |  | 4 | 21.9 | 24.623 | | |
| G5E604;G5E5H2;L8HK92 | Uncharacterized protein |  | 4 | 43.9 | 11.058 | | |
| **Buffalo** |  |  |  |  |  | | |
| P20757 | Angiotensinogen | AGT | 5 | 13.4 | 51.303 | | |
| L8ISP4 | Serum albumin | ALB | 26 | 52.2 | 69.337 | | |
| P04272;P19620;Q2Q1M6 | Annexin 2 | ANXA2 | 2 | 5.5 | 39.959 | | |
| Q3ZCH5;F1RNP2;L8IS19 | Zinc-alpha-2-glycoprotein | AZGP1 | 3 | 12.4 | 33.851 | | |
| A4ZVY8;A4ZVY9;Q6QAT4 | Beta-2-microglobulin | B2M | 6 | 65.3 | 13.609 | | |
| E9NRZ3;F5CC79 | Beta-1,4-galactosyltransferase I | B4GALT1 | 8 | 29.9 | 44.758 | | |
| P18892;L8HLI3 | Butyrophilin subfamily 1 member A1 | BTN1A1 | 17 | 42.8 | 59.276 | | |
| M9WLX8 | CD36 | CD36 | 5 | 14.4 | 52.742 | | |
| Q3ZCD0;L8I3A3;G3MYH4 | CD81 antigen | CD81 | 3 | 25 | 25.853 | | |
| P30932;L8HXM8;G8JKX6 | CD9 antigen | CD9 | 3 | 20.8 | 25.258 | | |
| A6QLC4;L8IKZ0;Q6R8F2 | Cadherin-1 | CDH1 | 2 | 2.2 | 97.974 | | |
| F1MX50;Q148D9 | Cellular repressor of E1A-stimulated genes 1 | CREG1 | 4 | 25.3 | 23.958 | | |
| Q3ZCL0;F6R3I5 | Cysteine-rich secretory protein 2 | CRISP3 | 6 | 36.5 | 27.453 | | |
| G3C8Y4;O62823;Q4F6X6 | Alpha-S1-casein | CSN1S1 | 13 | 86.9 | 24.326 | | |
| E9NZN2;B6VPY3 | Alpha-S2-casein | CSN1S2 | 20 | 62.2 | 26.118 | | |
| B7VGH4;Q9TSI0 | Beta casein | CSN2 | 8 | 47.3 | 25.106 | | |
| O02782 | Kappa-casein (Fragment) | CSN3 | 3 | 30.5 | 8.8378 | | |
| L8J0Y3;P01035 | Cystatin-C (Fragment) | CST3 | 4 | 45.7 | 16.651 | | |
| P80209;Q9BGU5 | Cathepsin D | CTSD | 4 | 12.6 | 44.704 | | |
| O46678;O97800 | Chemokine (C-X-C motif) ligand 2 | CXCL2 | 2 | 31.7 | 10.998 | | |
| G3MZ88;L8I0U1 | DnaJ-like protein subfamily B member 9 | DNAJB9 | 4 | 22 | 25.715 | | |
| Q3ZC71;L5JTX7 | Dolichol-phosphate mannosyltransferase subunit 3 | DPM3 | 2 | 19.8 | 12.169 | | |
| P10790;Q4TZH2 | Fatty acid-binding protein | FABP3 | 8 | 58.6 | 14.779 | | |
| F1N647;Q71SP7 | Fatty acid synthase | FASN | 28 | 16.4 | 274.26 | | |
| F1N6Y1;A6QNJ8 | Neutral alpha-glucosidase AB | GANAB | 11 | 14.6 | 109.47 | | |
| L8I6N0 | Glycosylation-dependent cell adhesion molecule 1 | GLYCAM1 | 3 | 15 | 17.114 | | |
| E1BHY6;Q2HJI6 | Granulin | GRN | 7 | 15 | 63.081 | | |
| E1BGX8;L8I229 | HHIP-like protein 2 (Fragment) | HHIPL2 | 11 | 16.3 | 81.178 | | |
| Q04967;F7A6V7 | Heat shock 70 kDa protein 6 | HSPA6 | 3 | 6.2 | 71.108 | | |
| Q9XSG3;Q0QEQ4 | Isocitrate dehydrogenase [NADP] cytoplasmic | IDH1 | 7 | 21.7 | 47.421 | | |
| A5D7Q2;L8HP94;G3MXB5 | Ig alpha-1 chain C region (Fragment) | IGHA1 | 8 | 26.1 | 51.67 | | |
| Q95M34 | Immunoglobulin gamma 1 heavy chain constant region (Fragment) | IGHC1 | 6 | 32.6 | 37.438 | | |
| H9GZN9 | Uncharacterized protein (Fragment) | IGHM | 16 | 50.4 | 49.438 | | |
| Q3SYR8 | Immunoglobulin J chain | IGJ | 3 | 21 | 17.857 | | |
| L8IAF4 | Ig lambda-1 chain C regions (Fragment) | IGLC1 | 5 | 74.3 | 11.185 | | |
| B1A4S9 | Alpha-lactalbumin | LALBA | 6 | 59.2 | 16.304 | | |
| E1B6Z6;L8HVH6 | Neutrophil gelatinase-associated lipocalin | LCN2 | 5 | 36.5 | 22.983 | | |
| Q6LBN7;G1CW23;Q19KS1 | Lactotransferrin | LF | 39 | 59.7 | 78.056 | | |
| C3W955;P02755 | Beta-lactoglobulin | LGB | 12 | 72.8 | 20.009 | | |
| P11151;Q29524 | Lipoprotein lipase | LPL | 10 | 28.9 | 53.591 | | |
| L8ICE9;P80025;G3MXZ0 | Lactoperoxidase | LPO | 18 | 34.6 | 80.611 | | |
| P80189;Q0MRP5 | Lysozyme | LYZ | 2 | 14.2 | 16.486 | | |
| L8HR34;M4QG36 | Lactadherin | MFGE8 | 20 | 53.8 | 47.875 | | |
| A7E340;E1BGW1;Q8MI01 | Mucin 15, cell surface associated | MUC15 | 3 | 9.4 | 35.685 | | |
| Q3MHX6;L5M9N2 | Protein OS-9 | OS9 | 2 | 4.5 | 75.806 | | |
| P81265;A6QNW3 | Polymeric immunoglobulin receptor | PIGR | 17 | 34.2 | 82.434 | | |
| A1L5C2;F1N1N6;Q9TUM6 | Perilipin-2 | PLIN2 | 15 | 45.6 | 49.307 | | |
| P62935;P62936 | Peptidyl-prolyl cis-trans isomerase | PPIA | 2 | 19.5 | 17.77 | | |
| L8I1I1;Q9BGI2 | Peroxiredoxin-4 | PRDX4 | 3 | 20.1 | 30.741 | | |
| A6QQA8;F1MM32;L8IAH9 | Sulfhydryl oxidase 1 | QSOX1 | 10 | 23.6 | 62.974 | | |
| Q9XSC9;L8HL52 | Transcobalamin-2 | TCN2 | 4 | 10.6 | 47.958 | | |
| P0CH28;L8IDB8;P0CG68 | Ubiquitin-40S ribosomal protein S27a | UBC | 4 | 56.1 | 77.569 | | |
| P80457 | Xanthine dehydrogenase/oxidase | XDH | 31 | 32.7 | 146.79 | | |
| G5E604;G5E5H2 | Uncharacterized protein |  | 4 | 43.9 | 11.058 | | |
| **Yak** |  |  |  |  |  | | |
| L8ISP4 | Serum albumin | ALB | 26 | 52.2 | 69.337 | |  |
| P10152;Q2NKV1;L8I8Q7 | Angiogenin-1 | ANG | 5 | 41.2 | 16.969 | |  |
| L8IVI5;Q2KJ51 | Angiopoietin-related protein 4 | ANGPTL4 | 2 | 5.1 | 45.537 | |  |
| F6Z2L5 | Uncharacterized protein | APOA1 | 7 | 31.2 | 30.33 | |  |
| Q3ZCH5;F1RNP2;L8IS19 | Zinc-alpha-2-glycoprotein | AZGP1 | 3 | 12.4 | 33.851 | |  |
| P01888;Q862Q3 | Beta-2-microglobulin | B2M | 5 | 55.9 | 13.677 | |  |
| E9NRZ3;F5CC79 | Beta-1,4-galactosyltransferase I | B4GALT1 | 8 | 29.9 | 44.758 | |  |
| P18892;L8HLI3 | Butyrophilin subfamily 1 member A1 | BTN1A1 | 17 | 42.8 | 59.276 | |  |
| L8HPZ1;P22226;P54230 | Cathelicidin-1 | CATHL1 | 6 | 43.9 | 17.631 | |  |
| P33046 | Cathelicidin-4 | CATHL4 | 3 | 31.2 | 16.479 | |  |
| M9WLX8 | CD36 | CD36 | 5 | 14.4 | 52.742 | |  |
| P30932;L8HXM8;G8JKX6 | CD9 antigen | CD9 | 3 | 20.8 | 25.258 | |  |
| A6QLC4;L8IKZ0;Q6R8F2 | Cadherin-1 | CDH1 | 2 | 2.2 | 97.974 | |  |
| F1MX50;Q148D9 | Cellular repressor of E1A-stimulated genes 1 | CREG1 | 4 | 25.3 | 23.958 | |  |
| Q3ZCL0;F6R3I5 | Cysteine-rich secretory protein 2 | CRISP3 | 6 | 36.5 | 27.453 | |  |
| A6QLZ7;D1Z306;L8IQ63 | Cysteine-rich secretory protein LCCL domain-containing 2 | CRISPLD2 | 4 | 9.9 | 55.581 | |  |
| L8I5S0;B5B3R8 | Alpha-S1-casein (Fragment) | CSN1S1 | 13 | 75.2 | 24.457 | |  |
| L8I6J3 | Alpha-S2-casein (Fragment) | CSN1S2 | 21 | 71.2 | 25.514 | |  |
| F5BHA8 | Beta-casein (Fragment) | CSN2 | 6 | 53.5 | 17.306 | |  |
| O02782 | Kappa-casein (Fragment) | CSN3 | 3 | 30.5 | 8.8378 | |  |
| L8J0Y3;P01035 | Cystatin-C (Fragment) | CST3 | 4 | 45.7 | 16.651 | |  |
| F1MMR6;L8I5C0;P80209 | Cathepsin D | CTSD | 4 | 12.6 | 44.704 | |  |
| L8HQV0;Q2KIS4;G1PIN8 | Dehydrogenase/reductase SDR family member 1 | DHRS1 | 2 | 9.6 | 34.023 | |  |
| G3MZ88;L8I0U1 | DnaJ-like protein subfamily B member 9 | DNAJB9 | 4 | 22 | 25.715 | |  |
| F1N036;Q27968 | DnaJ homolog subfamily C member 3 | DNAJC3 | 3 | 7.5 | 57.731 | |  |
| G1P1X5;G1MKK3;Q3ZC71 | Dolichol-phosphate mannosyltransferase subunit 3 | DPM3 | 2 | 19.8 | 12.169 | |  |
| P68103;Q0PY11;Q66RN5 | Elongation factor 1-alpha | EEF1A1 | 9 | 29.2 | 50.856 | |  |
| P10790;Q4TZH2;Q5XLB1 | Fatty acid-binding protein | FABP3 | 8 | 58.6 | 14.779 | |  |
| F7DDN1;F7CQT0 | Uncharacterized protein (Fragment) | FASN | 29 | 15 | 273.55 | |  |
| L8HY03 | Fibroblast growth factor-binding protein 1 | FGFBP1 | 2 | 10.7 | 26.183 | |  |
| Q3SZZ9;P12799 | Fibrinogen gamma-B chain | FGG | 5 | 16.5 | 50.231 | |  |
| Q0VCN9;Q9XSH1 | Folate receptor alpha | FOLR1 | 2 | 7.4 | 29.753 | |  |
| L8I0R2;I3LNH3;P79403 | Neutral alpha-glucosidase AB | GANAB | 11 | 14.6 | 109.47 | |  |
| L8I6N0 | Glycosylation-dependent cell adhesion molecule 1 | GLYCAM1 | 3 | 15 | 17.114 | |  |
| F1N726;L8IK54;Q0IIA4 | Glycoprotein 2 | GP2 | 12 | 26.6 | 59.248 | |  |
| Q1JPD9;D2HYX4 | G protein-coupled receptor, family C, group 5, member B | GPRC5B | 3 | 8.1 | 45.312 | |  |
| E1BHY6;Q2HJI6 | Granulin | GRN | 7 | 15 | 63.081 | |  |
| E1BGX8;L8I229 | HHIP-like protein 2 (Fragment) | HHIPL2 | 11 | 16.3 | 81.178 | |  |
| I6YLY8;P19120 | Heat shock cognate 71 kDa protein | HSPA8 | 7 | 16.9 | 71.238 | |  |
| A5D7Q2;L8HP94;G3MXB5 | Ig alpha-1 chain C region (Fragment) | IGHA1 | 8 | 26.1 | 51.67 | |  |
| L8HKR7 | Ig gamma-3 chain C region (Fragment) | IGHG3 | 4 | 19.4 | 33.279 | |  |
| H9GZN9 | Uncharacterized protein (Fragment) | IGHM | 16 | 50.4 | 49.438 | |  |
| Q3SYR8 | Immunoglobulin J chain | IGJ | 3 | 21 | 17.857 | |  |
| L8IAF4 | Ig lambda-1 chain C regions (Fragment) | IGLC1 | 5 | 74.3 | 11.185 | |  |
| L5L6W7;L5MAH8 | Keratin, type II cytoskeletal 75 | KRT75 | 8 | 11.4 | 60.758 | |  |
| B1A4S9 | Alpha-lactalbumin | LALBA | 6 | 59.2 | 16.304 | |  |
| F1MNN7;Q2TBI0 | Lipopolysaccharide-binding protein | LBP | 5 | 12.9 | 53.694 | |  |
| E1B6Z6;L8HVH6 | Neutrophil gelatinase-associated lipocalin | LCN2 | 5 | 36.5 | 22.983 | |  |
| B3VTM3;Q19KS1 | Lactotransferrin | LF | 39 | 59.7 | 78.056 | |  |
| L8J1Z0 | Beta-lactoglobulin | LGB | 12 | 73.6 | 19.811 | |  |
| Q95JH0;Q95JH2 | Lysophosphatidic acid acyltransferase | LPAAT | 2 | 14.6 | 32.071 | |  |
| A0FI82;P11151;Q29524 | Lipoprotein lipase | LPL | 10 | 28.9 | 53.591 | |  |
| L8ICE9;P80025;G3MXZ0 | Lactoperoxidase | LPO | 18 | 34.6 | 80.611 | |  |
| L8HR34;M4QG36 | Lactadherin | MFGE8 | 20 | 53.8 | 47.875 | |  |
| O18831;Q6SVB3;Q6UKZ8 | Myostatin | MSTN | 4 | 13.1 | 42.778 | |  |
| L8IYY3;L8J1Y8 | Odorant-binding protein 2b (Fragment) | OBP2B | 3 | 13.5 | 19.918 | |  |
| Q3MHX6;L5M9N2 | Protein OS-9 | OS9 | 2 | 4.5 | 75.806 | |  |
| P81265;A6QNW3 | Polymeric immunoglobulin receptor | PIGR | 17 | 34.2 | 82.434 | |  |
| A1L5C2;Q9TUM6 | Perilipin-2 | PLIN2 | 15 | 45.6 | 49.307 | |  |
| P62935;P62936 | Peptidyl-prolyl cis-trans isomerase | PPIA | 2 | 19.5 | 17.77 | |  |
| A6QQA8;F1MM32 | Sulfhydryl oxidase 1 | QSOX1 | 10 | 23.6 | 62.974 | |  |
| A5PJY7;L8I9S6 | Non-secretory ribonuclease | RNASE2 | 2 | 14.6 | 18.091 | |  |
| Q58DP6;L8I6U9;P15467 | Ribonuclease, RNase A family, 4 | RNASE4 | 5 | 50.3 | 16.938 | |  |
| A6QPW7;J9P6Y0 | Tumor necrosis factor receptor superfamily member 6B | TNFRSF6B | 2 | 7.1 | 32.375 | |  |
| P0CH28;L8IDB8;P0CG68 | Ubiquitin-40S ribosomal protein S27a | UBC | 4 | 56.1 | 77.569 | |  |
| P80457 | Xanthine dehydrogenase/oxidase | XDH | 31 | 32.7 | 146.79 | |  |
| G3N0V0 | Uncharacterized protein (Fragment) |  | 5 | 23 | 35.951 |  | |
| F1MLW8 | Uncharacterized protein |  | 4 | 21.9 | 24.623 | |  |
| G5E604;G5E5H2;L8HK92 | Uncharacterized protein |  | 4 | 43.9 | 11.058 | |  |
| **Goat** |  |  |  |  |  | |  |
| P68137;P68138 | Actin, alpha cardiac muscle 1 | ACTA1 | 8 | 24.1 | 42.975 | |  |
| P63258;O46546;Q9GLY0 | Actin, cytoplasmic 2 | ACTG1 | 13 | 47.1 | 42.273 | |  |
| G3EHG6;M9TKR5;A6ZE99 | Adipose differentiation-related protein | ADFP | 12 | 36 | 49.146 | |  |
| B3VHM9;P14639 | Albumin (Fragment) | ALB | 16 | 33.6 | 66.312 | |  |
| L8IVI5;Q2KJ51 | Angiopoietin-related protein 4 | ANGPTL4 | 2 | 5.1 | 45.537 | |  |
| P46193;Q6BCI9 | Annexin A1 | ANXA1 | 2 | 7.8 | 38.979 | |  |
| Q3ZCH5;F1RNP2;L8IS19 | Zinc-alpha-2-glycoprotein | AZGP1 | 3 | 12.4 | 33.851 | |  |
| G3N2H4;L8HQG0;G3N0Q8 | Azurocidin | AZU1 | 2 | 14.2 | 28.102 | |  |
| A4ZVY8;A4ZVY9;Q6QAT4 | Beta-2-microglobulin | B2M | 6 | 65.3 | 13.609 | |  |
| E9NRZ3;F5CC79 | Beta-1,4-galactosyltransferase I | B4GALT1 | 8 | 29.9 | 44.758 | |  |
| Q9XSQ9 | Bac7.5 protein | bac7.5 | 2 | 12.1 | 21.835 | |  |
| O02678;O46390;P21809 | Biglycan | BGN | 4 | 14 | 41.895 | |  |
| F7DU87;Q0PMN3 | Uncharacterized protein | BPIFA2 | 2 | 13.7 | 26.915 | |  |
| A3EY52 | Butyrophilin subfamily 1 member A1 | BTN1A1 | 15 | 37.6 | 59.265 | |  |
| L8HPZ1;P22226;P54230 | Cathelicidin-1 | CATHL1 | 6 | 43.9 | 17.631 | |  |
| P79362;P82018 | Cathelicidin-2 | CATHL2 | 3 | 21 | 19.842 | |  |
| F8U3U7 | Platelet glycoprotein 4 | CD36 | 3 | 9.5 | 52.831 | |  |
| Q3ZCD0;L8I3A3;G3MYH4 | CD81 antigen | CD81 | 3 | 25 | 25.853 | |  |
| P30932;L8HXM8;G8JKX6 | CD9 antigen | CD9 | 3 | 20.8 | 25.258 | |  |
| A6QLC4;L8IKZ0;Q6R8F2 | Cadherin-1 | CDH1 | 2 | 2.2 | 97.974 | |  |
| Q8SPQ0;Q7YS85;Q6TMG6 | Chitinase-3-like protein 1 | CHI3L1 | 3 | 11.2 | 42.893 | |  |
| F1MX50;Q148D9;F1S268 | Cellular repressor of E1A-stimulated genes 1 | CREG1 | 4 | 25.3 | 23.958 | |  |
| Q3ZCL0;F6R3I5 | Cysteine-rich secretory protein 2 | CRISP3 | 6 | 36.5 | 27.453 | |  |
| A6QLZ7;D1Z306;L8IQ63 | Cysteine-rich secretory protein LCCL domain-containing 2 | CRISPLD2 | 4 | 9.9 | 55.581 | |  |
| P18626 | Alpha-S1-casein | CSN1S1 | 14 | 67.8 | 24.289 | |  |
| P33049 | Alpha-S2-casein | CSN1S2 | 24 | 77.6 | 26.389 | |  |
| P33048;Q712N8;P11839 | Beta-casein | CSN2 | 11 | 56.8 | 24.865 | |  |
| P02670;Q540J1;Q547A4 | Kappa-casein | CSN3 | 10 | 47.4 | 21.441 | |  |
| K4P494 | Cystatin C | CST3 | 3 | 36.3 | 16.157 | |  |
| P80209;Q9BGU5 | Cathepsin D | CTSD | 4 | 12.6 | 44.704 | |  |
| Q2KIE5;F1SGI1 | Chemokine (C-X-C motif) ligand 2 | CXCL2 | 2 | 31.7 | 10.998 | |  |
| L8HQV0;Q2KIS4;G1PIN8 | Dehydrogenase/reductase SDR family member 1 | DHRS1 | 2 | 9.6 | 34.023 | |  |
| G3MZ88;L8I0U1 | DnaJ-like protein subfamily B member 9 | DNAJB9 | 4 | 22 | 25.715 | |  |
| Q27968;L8I378 | DnaJ homolog subfamily C member 3 | DNAJC3 | 3 | 7.5 | 57.731 | |  |
| L8IGM2;K9J179 | Dolichol-phosphate mannosyltransferase subunit 3 | DPM3 | 2 | 19.8 | 12.169 | |  |
| P68103;Q0PY11 | Elongation factor 1-alpha | EEF1A1 | 9 | 29.2 | 50.856 | |  |
| P10790;Q4TZH2 | Fatty acid-binding protein | FABP3 | 8 | 58.6 | 14.779 | |  |
| Q06B57;Q076H7 | Fatty acid synthase | FASN | 23 | 12 | 273.85 | |  |
| P07589;L8HQT5 | Fibronectin | FN1 | 16 | 9.7 | 272.12 | |  |
| F1N6Y1;A6QNJ8;P79403 | Neutral alpha-glucosidase AB | GANAB | 11 | 14.6 | 109.47 | |  |
| L8I6N0 | Glycosylation-dependent cell adhesion molecule 1 | GLYCAM1 | 3 | 15 | 17.114 | |  |
| Q1JPD9;K9J5D9;L8HX53 | G protein-coupled receptor, family C, group 5, member B | GPRC5B | 3 | 8.1 | 45.312 | |  |
| P37141;L5L1H2 | Glutathione peroxidase 3 | GPX3 | 2 | 11.5 | 25.681 | |  |
| E1BHY6;Q2HJI6 | Granulin | GRN | 7 | 15 | 63.081 | |  |
| P02077;Q1KZF3;P02076 | Hemoglobin subunit beta | HBBA | 4 | 40.7 | 16.021 | |  |
| E1BGX8;L8I229 | HHIP-like protein 2 (Fragment) | HHIPL2 | 11 | 16.3 | 81.178 | |  |
| Q6XUZ5;Q0QEQ3 | Isocitrate dehydrogenase [NADP] cytoplasmic | IDH1 | 7 | 21.7 | 47.421 | |  |
| A5D7Q2;L8HP94;G3MXB5 | Ig alpha-1 chain C region (Fragment) | IGHA1 | 8 | 26.1 | 51.67 | |  |
| Q95M34 | Immunoglobulin gamma 1 heavy chain constant region | IGHC1 | 6 | 32.6 | 37.438 | |  |
| Q3SYR8 | Immunoglobulin J chain | IGJ | 3 | 21 | 17.857 | |  |
| A5JSS8;P00712;P09462 | Alpha-lactalbumin | LALBA | 6 | 33.8 | 16.254 | |  |
| F1MNN7;Q2TBI0 | Lipopolysaccharide-binding protein | LBP | 5 | 12.9 | 53.694 | |  |
| E1B6Z6;L8HVH6 | Neutrophil gelatinase-associated lipocalin | LCN2 | 5 | 36.5 | 22.983 | |  |
| A3QPC1;Q29477 | Lactotransferrin | LF | 36 | 60.6 | 77.338 | |  |
| P02756;P67975;Q00P86 | Beta-lactoglobulin | LGB | 11 | 71.1 | 19.975 | |  |
| Q95JH0;Q95JH2 | Lysophosphatidic acid acyltransferase | LPAAT | 2 | 14.6 | 32.071 | |  |
| P11151;Q29524 | Lipoprotein lipase | LPL | 10 | 28.9 | 53.591 | |  |
| A3F9D6 | Lactoperoxidase | LPO | 12 | 21.9 | 80.339 | |  |
| C7FDJ1;G5CC03;G5CC04 | Milk fat globule-EGF factor 8 | MFGE8 | 16 | 48.4 | 47.444 | |  |
| O18831;Q6SVB3 | Myostatin | MSTN | 4 | 13.1 | 42.778 | |  |
| Q3MHX6;L5M9N2 | Protein OS-9 | OS9 | 2 | 4.5 | 75.806 | |  |
| B5T255;L8IR97 | Peptidoglycan recognition protein 1 | PGLYRP1 | 6 | 39.5 | 21.037 | |  |
| P81265;A6QNW3;P81265-2 | Polymeric immunoglobulin receptor | PIGR | 17 | 34.2 | 82.434 | |  |
| A1L5C2;F1N1N6;Q9TUM6 | Perilipin-2 | PLIN2 | 15 | 45.6 | 49.307 | |  |
| P62935;P62936 | Peptidyl-prolyl cis-trans isomerase | PPIA | 2 | 19.5 | 17.77 | |  |
| A6QQA8;F1MM32 | Sulfhydryl oxidase 1 | QSOX1 | 10 | 23.6 | 62.974 | |  |
| Q58DP6;L8I6U9;P15467 | Ribonuclease, RNase A family, 4 | RNASE4 | 5 | 50.3 | 16.938 | |  |
| A5JST2 | Serum amyloid A protein | SAA1 | 4 | 35.2 | 14.296 | |  |
| Q0PG40 | Serum amyloid A protein | SAA3 | 5 | 42.7 | 14.562 | |  |
| A9YUB7;Q9XSY9 | Osteopontin | SPP1 | 6 | 19.9 | 30.882 | |  |
| P0CG53;P0CG55 | Ubiquitin-40S ribosomal protein S27a | UBC | 4 | 56.1 | 77.569 | |  |
| A1YZ34 | Xanthine oxidoreductase | XDH | 21 | 19.4 | 146.98 | |  |
| F7BTW7;F7CJG3;F7E454 | Uncharacterized protein |  | 6 | 4.8 | 185.98 | |  |
| G3N0V0 | Uncharacterized protein (Fragment) |  | 5 | 23 | 35.951 | |  |
| **Camel** |  |  |  |  |  | |  |
| A7YWF1;Q19RL8 | Acetyl-coenzyme A synthetase, cytoplasmic | ACSS2 | 4 | 7.2 | 79.372 | |  |
| P68137;P68138 | Actin, alpha cardiac muscle 1 | ACTA1 | 8 | 24.1 | 42.975 | |  |
| Q9GLY0;Q06YY0 | Actin, cytoplasmic 2 | ACTG1 | 13 | 47.1 | 42.273 | |  |
| D0G0C3;Q710C4 | Adenosylhomocysteinase | AHCY | 3 | 9.5 | 47.724 | |  |
| F7BAY6;P35747;Q5XLE4 | Serum albumin | ALB | 22 | 43.2 | 68.372 | |  |
| A6QLL8;J9P7A6 | Fructose-bisphosphate aldolase | ALDOA | 2 | 2.7 | 74.896 | |  |
| L8IVI5;Q2KJ51 | Angiopoietin-related protein 4 | ANGPTL4 | 2 | 5.1 | 45.537 | |  |
| Q2QB47;M1ZMN6 | Aldehyde oxidase | AOH3 | 3 | 0.8 | 367.6 | |  |
| P84080;P84081 | ADP-ribosylation factor 3 | ARF1 | 2 | 5.4 | 73.212 | |  |
| Q3SZF2;Q56P20 | ADP-ribosylation factor 4 | ARF4 | 2 | 12.9 | 31.133 | |  |
| E9NRZ3;F5CC79 | Beta-1,4-galactosyltransferase I | B4GALT1 | 8 | 29.9 | 44.758 | |  |
| P18892;L8HLI3 | Butyrophilin subfamily 1 member A1 | BTN1A1 | 17 | 42.8 | 59.276 | |  |
| L5L3U6;Q2NKZ1 | T-complex protein 1 subunit eta | CCT7 | 2 | 7.7 | 62.5 | |  |
| F6U904;D5IGC7 | Platelet glycoprotein 4 | CD36 | 5 | 11.9 | 52.788 | |  |
| A7WLI1;Q007T3 | CD81 antigen | CD81 | 2 | 18.2 | 25.752 | |  |
| A6QLC4;L8IKZ0;Q6R8F2 | Cadherin-1 | CDH1 | 2 | 2.2 | 97.974 | |  |
| Q9XSA7;L5JUZ6 | Chloride intracellular channel protein 4 | CLIC4 | 2 | 10.3 | 28.714 | |  |
| P49951;L8IQH4 | Clathrin heavy chain 1 | CLTC | 3 | 2.3 | 192.86 | |  |
| Q29482;E2QYU2;P25473 | Clusterin | CLU | 4 | 10.7 | 52.153 | |  |
| F1MX50;Q148D9 | Cellular repressor of E1A-stimulated genes 1 | CREG1 | 4 | 25.3 | 23.958 | |  |
| Q28452 | Zeta-crystallin | CRYZ | 7 | 36.1 | 35.187 | |  |
| O97943;O97943-2;K7DXB9 | Alpha-S1-casein | CSN1S1 | 15 | 57 | 26.861 | |  |
| O97944 | Alpha-S2-casein | CSN1S2 | 13 | 54.4 | 22.964 | |  |
| M1E4K4;Q9TVD0 | Beta-casein | CSN2 | 7 | 54.7 | 26.174 | |  |
| P79139;Q28451 | Kappa casein | CSN3 | 6 | 35.7 | 20.373 | |  |
| Q3ZC71;L5JTX7 | Dolichol-phosphate mannosyltransferase subunit 3 | DPM3 | 2 | 19.8 | 12.169 | |  |
| Q864M1;Q866G8 | Elongation factor 1-alpha | EEF1A1 | 9 | 29.2 | 50.856 | |  |
| Q3SYU2;M1EPB6 | Elongation factor 2 | EEF2 | 3 | 4.2 | 95.585 | |  |
| L5LRC3;A6QR19 | Alpha-enolase | ENO1 | 2 | 7.6 | 47.339 | |  |
| L5KEG8;F7BPT4 | Uncharacterized protein | EZR | 7 | 16.4 | 69.448 | |  |
| P10790;Q4TZH2 | Fatty acid-binding protein | FABP3 | 8 | 58.6 | 14.779 | |  |
| F7DDN1;F7CQT0 | Uncharacterized protein (Fragment) | FASN | 29 | 15 | 273.55 | |  |
| F6RUZ6;F1PBL4 | Fibrinogen alpha chain | FGA | 2 | 2.8 | 87.775 | |  |
| Q9MZ06;Q4VS17 | Fibroblast growth factor-binding protein 1 | FGFBP1 | 5 | 18.8 | 26.188 | |  |
| Q3SZZ9;P12799 | Fibrinogen gamma-B chain | FGG | 5 | 16.5 | 50.231 | |  |
| P07589;L8HQT5 | Fibronectin | FN1 | 16 | 9.7 | 272.12 | |  |
| Q2MHN2;P18685 | Ferritin heavy chain | FTH1 | 4 | 26.8 | 21.308 | |  |
| A6QNJ8;P79403 | Neutral alpha-glucosidase AB | GANAB | 11 | 14.6 | 109.47 | |  |
| P10096;Q28259 | Glyceraldehyde-3-phosphate dehydrogenase | GAPDH | 6 | 26.7 | 35.82 | |  |
| Q0VCH9;J9NX46 | Golgi-associated plant pathogenesis-related protein 1 | GLIPR2 | 3 | 16.3 | 31.447 | |  |
| P15522;P15522-2 | Glycosylation-dependent cell adhesion molecule 1 | GLYCAM1 | 8 | 52.3 | 17.291 | |  |
| P62871;P62872 | Guanine nucleotide binding protein , beta polypeptide 1 | GNB1 | 3 | 14.2 | 40.159 | |  |
| F7AB03;K9ILK6 | Uncharacterized protein (Fragment) | GRN | 2 | 4.8 | 64.91 | |  |
| F1Q0J0;P80031 | Glutathione S-transferase Pi class | GSTP1 | 2 | 16.7 | 23.529 | |  |
| F7DX75 | HAPLN3 protein | HAPLN3 | 8 | 32.6 | 31.4 | |  |
| F6TAH8 | Uncharacterized protein (Fragment) | HHIPL2 | 6 | 8 | 83.105 | |  |
| O02705;Q76LV2 | Heat shock protein alpha | HSP90AA1 | 6 | 9.7 | 86.076 | |  |
| Q76LV1;Q9GKX8 | Heat shock protein HSP 90-beta | HSP90AB1 | 7 | 10.5 | 85.135 | |  |
| Q27965;P34930 | Heat shock protein 70 (Fragment) | HSPA1B | 6 | 11.9 | 78.087 | |  |
| Q04967;L8I314 | Heat shock 70 kDa protein 6 | HSPA6 | 3 | 6.2 | 71.108 | |  |
| P19120;K9J3G1 | Heat shock cognate 71 kDa protein | HSPA8 | 7 | 16.9 | 71.238 | |  |
| Q9XSG3;Q0QEQ4 | Isocitrate dehydrogenase [NADP] cytoplasmic | IDH1 | 7 | 21.7 | 47.421 | |  |
| L8IAF4 | Ig lambda-1 chain C regions (Fragment) | IGLC1 | 5 | 74.3 | 11.185 | |  |
| F7CWC8 | L-amino-acid oxidase | IL4I1 | 22 | 50.2 | 56.944 | |  |
| P00710 | Alpha-lactalbumin | LALBA | 7 | 52.8 | 14.43 | |  |
| F1MNN7;Q2TBI0 | Lipopolysaccharide-binding protein | LBP | 5 | 12.9 | 53.694 | |  |
| P00336;P00339 | L-lactate dehydrogenase | LDHB | 2 | 6.5 | 37.161 | |  |
| Q9TUM0 | Lactotransferrin | LF | 31 | 60.3 | 77.211 | |  |
| P11151;Q29524 | Lipoprotein lipase | LPL | 10 | 28.9 | 53.591 | |  |
| Q9GJW6 | Peroxidase | LPO | 13 | 23.2 | 80.674 | |  |
| P27424;F6VPC9 | Microfibrillar-associated protein 2 | MFAP2 | 2 | 9.6 | 21.142 | |  |
| F5BZ34 | Milk fat globule EGF factor 8 (Fragment) | MFGE8 | 13 | 46.3 | 32.786 | |  |
| L5JVC6;Q2HJ49 | Moesin | MSN | 9 | 13.3 | 78.895 | |  |
| O18831;Q6SVB3 | Myostatin | MSTN | 4 | 13.1 | 42.778 | |  |
| A7E340;Q8MI01 | Mucin 15, cell surface associated | MUC15 | 3 | 9.4 | 35.685 | |  |
| Q9GK12 | Peptidoglycan recognition protein 1 | PGLYRP1 | 5 | 39.9 | 21.377 | |  |
| P81265;A6QNW3 | Polymeric immunoglobulin receptor | PIGR | 17 | 34.2 | 82.434 | |  |
| Q9TUM6Q8WNR8 | Perilipin-2 | PLIN2 | 15 | 45.6 | 49.307 | |  |
| P62935;P62936 | Peptidyl-prolyl cis-trans isomerase | PPIA | 2 | 19.5 | 17.77 | |  |
| ;P80311;Q5TIK9 | Peptidyl-prolyl cis-trans isomerase | PPIB | 3 | 15.8 | 26.437 | |  |
| A6QQA8;L8IAH9 | Sulfhydryl oxidase 1 | QSOX1 | 10 | 23.6 | 62.974 | |  |
| L5K969;Q3MHP2 | Ras-related protein Rab-11B | RAB11B | 2 | 13.3 | 25.204 | |  |
| Q0IIG8;G9KJR0 | Ras-related protein Rab-18 | RAB18 | 3 | 20.5 | 26.935 | |  |
| P62822;Q52NJ2 | Ras-related protein Rab-1A (Fragment) | RAB1A | 5 | 28.3 | 27.157 | |  |
| Q06AU7;Q2HJH2 | RAB1B, member RAS oncoprotein family (Fragment) | RAB1B | 5 | 34.6 | 23.458 | |  |
| P24406;P61585 | Transforming protein RhoA-like protein | RHOA | 3 | 27.3 | 21.896 | |  |
| Q58DP6;L8I6U9;P15467 | Ribonuclease, RNase A family, 4 | RNASE4 | 5 | 50.3 | 16.938 | |  |
| Q29214;Q95140 | 60S acidic ribosomal protein P0 | RPLP0 | 2 | 1.1 | 327.33 | |  |
| P42899;Q6X9Z5 | 60S acidic ribosomal protein P2 (Fragment) | RPLP2 | 2 | 10.6 | 15.02 | |  |
| Q9XSU9;Q66TU2;L5LZ44 | 40S ribosomal protein S14 (Fragment) | RPS14 | 2 | 14.8 | 17.602 | |  |
| P62272;Q3T0R1 | 40S ribosomal protein S18 | RPS18 | 2 | 12.8 | 18.782 | |  |
| Q32PD5;Q29308 | 40S ribosomal protein S19 | RPS19 | 2 | 4.2 | 56.911 | |  |
| Q0Z8U2;Q3T169 | 40S ribosomal protein S3 | RPS3 | 2 | 8.4 | 27.519 | |  |
| P26452;Q4GWZ2 | 40S ribosomal protein SA | RPSA | 3 | 5.4 | 91.669 | |  |
| F6ZTL0 | Sodium/nucleoside cotransporter | SLC28A3 | 8 | 12.9 | 73.651 | |  |
| F1S5A6;H2EJJ3 | Type IIb Na-dependent phosphate cotransporter | SLC34A2 | 2 | 2.7 | 73.7 | |  |
| Q3SZK8;L5KP51 | Solute carrier family 9 (Sodium/hydrogen exchanger) member 3 regulator 1 | SLC9A3R1 | 8 | 24.7 | 39.304 | |  |
| A6QQN6;F6ULX5 | Acid sphingomyelinase-like phosphodiesterase 3b | SMPDL3B | 2 | 6.4 | 50.905 | |  |
| Q9XSC9;L8HL52 | Transcobalamin-2 | TCN2 | 4 | 10.6 | 47.958 | |  |
| P0CG53;P0CG55 | Ubiquitin-40S ribosomal protein S27a | UBC | 4 | 56.1 | 77.569 | |  |
| P79303;Q07130 | UTP--glucose-1-phosphate uridylyltransferase | UGP2 | 4 | 10.7 | 58.521 | |  |
| Q3T0Y8;G1P4K2 | Vesicle-associated membrane protein 8 | VAMP8 | 2 | 15.6 | 14.42 | |  |
| P09837 | Whey acidic protein | WAP | 3 | 30.8 | 12.564 | |  |
| Q2KJH4;F6Z8W0 | WD repeat-containing protein 1 | WDR1 | 2 | 2.7 | 68.324 | |  |
| F7D8I6 | Xanthine dehydrogenase/oxidase-like protein | XDH | 31 | 30.8 | 146.67 | |  |
| P68250;P68251 | 14-3-3 protein beta/alpha | YWHAB | 4 | 11.4 | 46.695 | |  |
| P62261;P62262 | 14-3-3 protein epsilon | YWHAE | 6 | 18.8 | 41.418 | |  |
| L5KR87;A4IE76 | 14-3-3 protein theta | YWHAQ | 5 | 5.7 | 125.08 | |  |
| A0SNV3;Q06YX6 | 14-3-3 protein zeta/delta | YWHAZ | 7 | 34.5 | 29.364 | |  |
| H9GZT5 | Uncharacterized protein (Fragment) |  | 8 | 26.3 | 36.432 | |  |
| **Horse** |  |  |  |  |  | |  |
| F6RI47;F6R942 | Alpha-2-macroglobulin | A2M | 15 | 12.6 | 164.17 | |  |
| Q19RL8;L5LU72;G1LRW3 | Acetyl-coenzyme A synthetase, cytoplasmic | ACSS2 | 4 | 7.2 | 79.372 | |  |
| P68137;P68138 | Actin, alpha cardiac muscle 1 | ACTA1 | 8 | 24.1 | 42.975 | |  |
| F6T3Y8 | Actin, cytoplasmic 1 (Fragment) | ACTB | 12 | 49.3 | 40.673 | |  |
| P63258;K9J2K5;A7UDB4 | Actin, cytoplasmic 2 | ACTG1 | 13 | 47.1 | 42.273 | |  |
| F7BAY6;P35747;Q5XLE4 | Serum albumin | ALB | 22 | 43.2 | 68.372 | |  |
| A6QLL8;J9P7A6 | Fructose-bisphosphate aldolase | ALDOA | 2 | 2.7 | 74.896 | |  |
| L5JZQ8;E1BBX5;F1PI09 | Aldehyde oxidase | AOH3 | 3 | 0.8 | 367.6 | |  |
| F6Z2L5 | Uncharacterized protein | APOA1 | 7 | 31.2 | 30.33 | |  |
| F6RM73;P83704 | Apolipoprotein A-II | APOA2 | 2 | 19 | 11.289 | |  |
| P84080;P84081;Q5E9I6 | ADP-ribosylation factor 3 | ARF1 | 2 | 5.4 | 73.212 | |  |
| Q3ZCH5;F1RNP2;L8IS19 | Zinc-alpha-2-glycoprotein | AZGP1 | 3 | 12.4 | 33.851 | |  |
| P30441;Q861S3;Q863A8 | Beta-2-microglobulin | B2M | 2 | 16.9 | 13.426 | |  |
| E9NRZ3;F5CC79 | Beta-1,4-galactosyltransferase I | B4GALT1 | 8 | 29.9 | 44.758 | |  |
| F7DU87;Q0PMN3 | Uncharacterized protein | BPIFA2 | 2 | 13.7 | 26.915 | |  |
| P18892;L8HLI3 | Butyrophilin subfamily 1 member A1 | BTN1A1 | 17 | 42.8 | 59.276 | |  |
| P01025 | Complement C3 | C3 | 9 | 6.5 | 186.8 | |  |
| L8HPZ1;P22226;P54230 | Cathelicidin-1 | CATHL1 | 6 | 43.9 | 17.631 | |  |
| F6U904;D5IGC7 | Platelet glycoprotein 4 | CD36 | 5 | 11.9 | 52.788 | |  |
| F6XGE0;F6XBQ7 | Uncharacterized protein | CD55 | 4 | 11.7 | 54.937 | |  |
| F7BXD8 | Fc receptor-like protein 2 | CD5L | 3 | 11.5 | 36.39 | |  |
| F6WQJ4 | Uncharacterized protein (Fragment) | CD9 | 2 | 20.6 | 22.594 | |  |
| F6VF11;E2RAB0;M3W937 | Chordin-like protein 2 | CHRDL2 | 6 | 18.7 | 47.44 | |  |
| F6ZIJ1 | Uncharacterized protein | CIDEA | 2 | 9.7 | 26.746 | |  |
| Q9XSA7;L5JUZ6 | Chloride intracellular channel protein 4 | CLIC4 | 2 | 10.3 | 28.714 | |  |
| Q29482;E2QYU2;P25473 | Clusterin | CLU | 4 | 10.7 | 52.153 | |  |
| Q148D9;F1S268;L8IQM1 | Cellular repressor of E1A-stimulated genes 1 | CREG1 | 4 | 25.3 | 23.958 | |  |
| F7DGH6;Q8SPR1;C3W972 | Alpha s1 casein | CSN1S1 | 8 | 48.1 | 25.158 | |  |
| D2KAS0 | AlphaS2-casein | CSN1S2 | 17 | 72.9 | 25.459 | |  |
| Q9GKK3;P86273 | Beta-casein | CSN2 | 8 | 48.1 | 27.049 | |  |
| P82187 | Kappa-casein | CSN3 | 8 | 38.9 | 21.021 | |  |
| P80209;Q9BGU5 | Cathepsin D | CTSD | 4 | 12.6 | 44.704 | |  |
| F7CXT0 | Uncharacterized protein (Fragment) | CYBRD1 | 2 | 11.3 | 24.497 | |  |
| Q3ZC71;L5JTX7 | Dolichol-phosphate mannosyltransferase subunit 3 | DPM3 | 2 | 19.8 | 12.169 | |  |
| Q864M1;Q866G8 | Elongation factor 1-alpha | EEF1A1 | 9 | 29.2 | 50.856 | |  |
| L5KEG8;F7BPT4 | Uncharacterized protein | EZR | 7 | 16.4 | 69.448 | |  |
| P10790;Q4TZH2 | Fatty acid-binding protein | FABP3 | 8 | 58.6 | 14.779 | |  |
| F7DDN1;F7CQT0 | Uncharacterized protein (Fragment) | FASN | 29 | 15 | 273.55 | |  |
| F6RUZ6;F1PBL4 | Fibrinogen alpha chain | FGA | 2 | 2.8 | 87.775 | |  |
| F6THP3 | Uncharacterized protein (Fragment) | FGFBP1 | 3 | 36.4 | 12.167 | |  |
| F6SUZ2;Q3T105 | UDP-glucose 4-epimerase | GALE | 2 | 10 | 38.468 | |  |
| A6QNJ8;P79403 | Neutral alpha-glucosidase AB | GANAB | 11 | 14.6 | 109.47 | |  |
| P10096;Q28259 | Glyceraldehyde-3-phosphate dehydrogenase | GAPDH | 6 | 26.7 | 35.82 | |  |
| F7DXY0 | Uncharacterized protein | GGT1 | 2 | 4.6 | 60.849 | |  |
| P62871;P62872 | Guanine nucleotide binding protein , beta polypeptide 1 | GNB1 | 3 | 14.2 | 40.159 | |  |
| L8IJ59;Q28024 | Guanine nucleotide-binding protein subunit gamma | GNG12 | 2 | 37.5 | 7.9921 | |  |
| F7AB03;K9ILK6 | Uncharacterized protein (Fragment) | GRN | 2 | 4.8 | 64.91 | |  |
| F7DX75 | HAPLN3 protein | HAPLN3 | 8 | 32.6 | 31.4 | |  |
| A5A8V6;Q6S4N2 | Heat shock protein 70 (Fragment) | HSPA1B | 6 | 11.9 | 78.087 | |  |
| Q04967;F7A6V7 | Heat shock 70 kDa protein 6 | HSPA6 | 3 | 6.2 | 71.108 | |  |
| P19120;K9J3G1 | Heat shock cognate 71 kDa protein | HSPA8 | 7 | 16.9 | 71.238 | |  |
| Q0QEQ6;Q0QEQ2 | Isocitrate dehydrogenase [NADP] cytoplasmic | IDH1 | 5 | 9 | 76.177 | |  |
| Q95M34 | Immunoglobulin gamma 1 heavy chain constant region (Fragment) | IGHC1 | 6 | 32.6 | 37.438 | |  |
| H9GZN9 | Uncharacterized protein (Fragment) | IGHM | 16 | 50.4 | 49.438 | |  |
| Q3SYR8 | Immunoglobulin J chain | IGJ | 3 | 21 | 17.857 | |  |
| F6V5H1 | Uncharacterized protein (Fragment) | IGKC | 3 | 42.3 | 11.245 | |  |
| F7CWC8 | L-amino-acid oxidase | IL4I1 | 22 | 50.2 | 56.944 | |  |
| F6YRC5;K9KDK9 | Ras GTPase-activating-like protein IQGAP1 | IQGAP1 | 2 | 2.3 | 187.12 | |  |
| F6V8C8;P28546 | Alpha-lactalbumin | LALBA | 8 | 65.7 | 16.333 | |  |
| F6XLB1;O77811 | Lactotransferrin | LF | 34 | 57.1 | 77.36 | |  |
| P02758 | Beta-lactoglobulin-1 | LGB1 | 10 | 62.8 | 20.344 | |  |
| P07380 | Beta-lactoglobulin-2 | LGB2 | 4 | 24.3 | 20.123 | |  |
| P11151;Q29524 | Lipoprotein lipase | LPL | 10 | 28.9 | 53.591 | |  |
| F6PPX7;P11376 | Lysozyme C, milk isozyme | LYZ | 5 | 37.8 | 16.799 | |  |
| F5CEP2 | Milk fat globule-EGF factor 8 splice variant (Fragment) | MFGE8 | 14 | 51 | 43.308 | |  |
| O18831;Q6SVB3 | Myostatin | MSTN | 4 | 13.1 | 42.778 | |  |
| F7BL38 | Uncharacterized protein (Fragment) | MUC1 | 4 | 24.7 | 18.819 | |  |
| F6VIZ0 | Mucin-4 | MUC4 | 12 | 8.1 | 227.3 | |  |
| F6XEP5;G1PE23 | Uncharacterized protein (Fragment) | NT5E | 3 | 7.8 | 50.509 | |  |
| D0V860;F6VVI1 | Amplified in osteosarcoma 9 | OS9 | 2 | 4.5 | 75.781 | |  |
| F6W1N4;F6W2K5 | Uncharacterized protein | PIGR | 22 | 38.8 | 83.003 | |  |
| F7D2U1;L5KK92 | Uncharacterized protein | PLBD1 | 2 | 4.5 | 63.841 | |  |
| F6Q9R1;K9KAB0 | Perilipin | PLIN2 | 12 | 42.7 | 45.758 | |  |
| P62935;P62936 | Peptidyl-prolyl cis-trans isomerase | PPIA | 2 | 19.5 | 17.77 | |  |
| A6QQA8;F1MM32 | Sulfhydryl oxidase 1 | QSOX1 | 10 | 23.6 | 62.974 | |  |
| A9LM14;P62490;Q2TA29 | Ras-related protein Rab-11B | RAB11B | 2 | 13.3 | 25.204 | |  |
| L5LW97;Q0IIG8 | Ras-related protein Rab-18 | RAB18 | 3 | 20.5 | 26.935 | |  |
| A1L528;A9LM10 | Ras-related protein Rab-1A (Fragment) | RAB1A | 5 | 28.3 | 27.157 | |  |
| F6PJF6 | Serum amyloid A protein | SAA1 | 5 | 36.9 | 14.488 | |  |
| F7CW51;L5JVJ3 | Syntenin-1 | SDCBP | 3 | 19.1 | 32.576 | |  |
| F6ZTL0 | Sodium/nucleoside cotransporter | SLC28A3 | 8 | 12.9 | 73.651 | |  |
| F6ZU69 | Uncharacterized protein | SLC34A2 | 9 | 17 | 75.415 | |  |
| Q9BDF6;M3YMU7;Q5U9D0;B3RF78 | Na+/glucose co-transporter | SLC5A1 | 2 | 2.7 | 73.05 | |  |
| F6WG98;K9IMZ2 | Transporter | SLC6A14 | 3 | 5.5 | 71.927 | |  |
| F7C7M0;F7C1X7 | Uncharacterized protein | SLC9A3R1 | 3 | 9.9 | 39.657 | |  |
| F7AYC1 | Uncharacterized protein | SPP1 | 9 | 39 | 35.019 | |  |
| P0CH28;L8IDB8;P0CG68 | Ubiquitin-40S ribosomal protein S27a | UBC | 4 | 56.1 | 77.569 | |  |
| P79303;Q07130 | UTP--glucose-1-phosphate uridylyltransferase | UGP2 | 4 | 10.7 | 58.521 | |  |
| F7D8I6 | Xanthine dehydrogenase/oxidase-like protein | XDH | 31 | 30.8 | 146.67 | |  |
| P62261;P62262 | 14-3-3 protein epsilon | YWHAE | 6 | 18.8 | 41.418 | |  |
| P63103;A0SNV3;Q06YX6 | 14-3-3 protein zeta/delta | YWHAZ | 7 | 34.5 | 29.364 | |  |
| H9GZT5 | Uncharacterized protein (Fragment) |  | 8 | 26.3 | 36.432 | |  |
| F7CAC5 | Uncharacterized protein (Fragment) |  | 3 | 28.2 | 12.231 | |  |
| F6SP11 | Uncharacterized protein (Fragment) |  | 5 | 62.3 | 10.98 | |  |
| H9GZU9 | Uncharacterized protein (Fragment) |  | 8 | 41.8 | 35.84 | |  |
| H9GZU8 | Uncharacterized protein (Fragment) |  | 6 | 27.5 | 35.915 | |  |
| F6TIR2 | Uncharacterized protein |  | 3 | 27.1 | 23.147 | |  |
| H9GZR2;H9GZS6 | Uncharacterized protein (Fragment) |  | 4 | 19.2 | 36.871 | |  |
| F6SQD7 | Uncharacterized protein (Fragment) |  | 3 | 40.2 | 11.226 | |  |
| H9GZV1 | Uncharacterized protein (Fragment) |  | 9 | 46.4 | 35.625 | |  |
| F7BTW7;F7CJG3;F7E454 | Uncharacterized protein |  | 6 | 4.8 | 185.98 | |  |
| F6QAU5 | Uncharacterized protein (Fragment) |  | 3 | 26.2 | 11.44 | |  |
| F6USP9 | Uncharacterized protein (Fragment) |  | 2 | 2.9 | 91.714 | |  |

Table S2 Information of the identified proteins by co-immunoprecipitation experiment.

| Accession No. | Protein name | Gene name | Mol. Weight (kDa) | Sample 1 | | Sample 2 | | | Sample 3 | | |
| --- | --- | --- | --- | --- | --- | --- | --- | --- | --- | --- | --- |
|  |  |  |  | Peptide | Score | | Peptide | Score | | Peptide | Score |
| P02755 | Beta-lactoglobulin | LGB | 19.870 | 24 | 100.3142 | | 22 | 90.31807 | | 53 | 120.32 |
| P24627 | Lactotransferrin | LTF | 78.006 | 12 | 100.2562 | | 5 | 50.24256 | | 19 | 150.2241 |
| P00711 | Alpha-lactalbumin | LALBA | 16.236 | 27 | 90.28377 | | 26 | 100.2846 | | 63 | 80.27507 |
| G5E5T5 | Uncharacterized protein |  | 42.445 | 9 | 70.27311 | | 4 | 30.20973 | | 15 | 80.27256 |
| P02668 | Kappa-casein | CSN3 | 21.255 | 8 | 50.30537 | | 3 | 20.30482 | | 4 | 20.25954 |
| G5E513 | Uncharacterized protein |  | 49.938 | 5 | 50.27057 | | 4 | 40.29491 | | 9 | 60.35384 |
| P81265 | Polymeric immunoglobulin receptor | PIGR | 82.383 | 6 | 50.25764 | | 5 | 50.22861 | | 11 | 80.28485 |
| P02769 | Serum albumin | ALB | 69.248 | 8 | 50.24648 | | 8 | 40.23858 | | 15 | 120.2505 |
| P02662 | Alpha-S1-casein | CSN1S1 | 24.513 | 9 | 50.23011 | | 8 | 40.22694 | | 12 | 60.26335 |
| A5PK72 | Uncharacterized protein |  | 24.790 | 7 | 40.23349 | | 8 | 30.21041 | | 13 | 40.26943 |
| P02666 | Beta-casein | CSN2 | 25.091 | 10 | 30.2788 | | 8 | 40.27531 | | 12 | 40.29947 |
| P17697 | Clusterin | CLU | 51.081 | 3 | 30.23985 | | 2 | 20.27869 | |  |  |
| P02663 | Alpha-S2-casein | CSN1S2 | 26.002 | 2 | 20.19466 | |  |  | | 3 | 20.2271 |
| Q2UVX4 | Complement C3 | C3 | 187.134 |  |  | | 2 | 20.19572 | | 5 | 50.28519 |
